# Photoactivatable immunomodulator polyprodrugs for boosting synergistic antitumor immunity of STING agonists and IDO inhibitors

**DOI:** 10.7150/thno.107774

**Published:** 2025-03-08

**Authors:** Guopu Huang, Cheng Li, Jiale Si, Yufei Cao, Moujiang Zheng, Yueming Xue, Qinghao Zhou, Zhishen Ge, Yuanyuan Ji

**Affiliations:** 1 Department of Geriatric General Surgery, The Second Affiliated Hospital, Xi'an Jiaotong University, Xi'an 710004, Shaanxi, China; 2 Scientific Research Center and Precision Medical Institute, The Second Affiliated Hospital, Xi'an Jiaotong University, Xi'an 710004, China; 3 School of Chemistry, Xi'an Key Laboratory of Sustainable Polymer Materials, Xi'an Jiaotong University, Xi'an 710049, Shaanxi, China

**Keywords:** IDO inhibitor, STING agonist, immunotherapy, photodynamic therapy, polymeric micelles

## Abstract

**Rationale:** Stimulator of interferon genes (STING) activation within tumors can inevitably enhance the activity of indoleamine 2,3-dioxygenase (IDO). However, IDO will convert tryptophan (Trp) into kynurenine (Kyn), which can inhibit Trp-sensitive T cells functional activity and induce immunosuppressive effects. The efficient nanomedicines for combination of STING agonist and IDO inhibitor have been rarely explored.

**Methods:** A diblock polymer polyprodrug was synthesized with the IDO inhibitor 1-methyl-tryptophan (1-MT) linked by thioketal bonds and the photosensitizer 5,10,15,20-tetraphenylporphyrin (TPP) in the hydrophobic block as well as endoplasmic reticulum (ER) targeting group (4-methylphenyl) sulfonamide in the hydrophilic block. After self-assembly in aqueous solution, the micelles loading STING agonist SR-717 (SR@ET-PMT) can be formed with a high loading efficiency. After cellular internalization, the micelles can target ER. Upon exposure to light irradiation of 650 nm, reactive oxygen species (ROS) can be generated to break thioketal bonds and dissociate the micelles to release 1-MT and STING agonist. Accompanied by photodynamic therapy (PDT), STING activation and IDO inhibition are achieved simultaneously.

**Results:** *In vitro* observation reveals the PDT effect, ER targeting, and photoactivated drug release. *In vivo* animal model results demonstrate that the photoactivatable immunomodulator polyprodrug micelles show excellent tumor accumulation and potent immune activation capability to inhibit solid tumors. The PDT effect, STING activation, and IDO inhibition synergistically activate *in vivo* antitumor immunity. Finally, SR@ET-PMT can attain an 88% suppression rate of solid tumors due to the potent immunotherapeutic efficacy.

**Conclusion:** The photoactivatable immunomodulator polyprodrugs are successfully prepared to simultaneously deliver STING agonists and IDO inhibitors, which represent a promising nanomedicine for the spatiotemporal activation of synergistic antitumor immunity.

## Introduction

Cancer immunotherapy has become a popular therapeutic modality of tumors because it can not only suppress tumors by stimulating the immune system but also elicit sustained immune responses to inhibit tumor recurrence 1-3. Among various immunotherapy methods, stimulator of interferon genes (STING) activation has emerged as one of most promising methods. Notably, endogenous cyclic dinucleotides (CDNs) cause conformational changes of STING dimers in the endoplasmic reticulum (ER) 4-8. Subsequently, the translocation of STING proteins into Golgi apparatus to collect and activate TANK-binding kinase 1 (TBK1) which further phosphorylates interferon regulatory factor 3 (IRF3). The phosphorylated IRF3 translocate to the nucleus for upregulation of immune type I interferon (IFN) expression. Type I IFNs can promote the maturation and migration of immune cells 9-11. However, STING activation and the interferon-γ (IFN-γ) that is secreted by cytotoxic T lymphocytes (CTLs) can also increase the activity of indoleamine 2,3-dioxygenase (IDO) 12-19. IDO will convert tryptophan (Trp) into kynurenine (Kyn), and the sustained consumption of Trp and accumulation of Kyn elicit severe immunosuppressive effects 15, 20-22. Thus, the combination of IDO inhibition and STING activation is expected to achieve synergistic antitumor effects, which has been rarely explored.

STING agonists including CDNs, diABZI 23, SR-717 24, MSA-2 25, and metal ions (Zn^2+^ and Mn^2+^) 26-28 have garnered considerable attention with remarkable antitumor therapeutic effects. It is well-known that the site of action for STING agonists is ER inside cells. Thus, the STING agonist must overcome a series of physiological barriers for efficient STING activation 10. However, these small molecule STING agonists commonly encounter issues such as poor water solubility, limited cytoplasmic entry, and difficulty for localization at ER. The delivery nanocarriers have been widely developed for STING agonist delivery 8, 28-36. As for IDO inhibitors including 1-methyl-tryptophan (1-MT) and NLG919 have gained wide attention for cancer immunotherapy 15, 37-45. However, they often grapple with the issues including off-target effects, inadequate targeting, and poor tumor retention 46-48. To address these challenges, various stimuli-responsive prodrug delivery systems have been developed and the endogenous stimuli including enzymes 38, 49, reactive oxygen species (ROS) 43, 44, 50, glutathione (GSH) 15, 39, have been employed as the triggers. In addition to the endogenous triggers, the external stimuli (e.g. light) with the characteristics of spatiotemporal controllability have become a very common trigger for responsive drug release. Photoactivated polyprodrugs have the distinct advantages including high drug loading efficiency and stability, precise control of drug release at specific sites and times, thereby minimizing off-target effects 51-54. Photoactivated polyprodrugs have been applied to the delivery of IDO inhibitors, which is a promising therapeutic strategy with potential clinical applications 43, 44. Despite of the great progresses, for combination of IDO inhibition and STING activation, effective simultaneous delivery of IDO inhibitors and STING agonists with the different structural features remains a great challenge 45.

**Figure 1:**
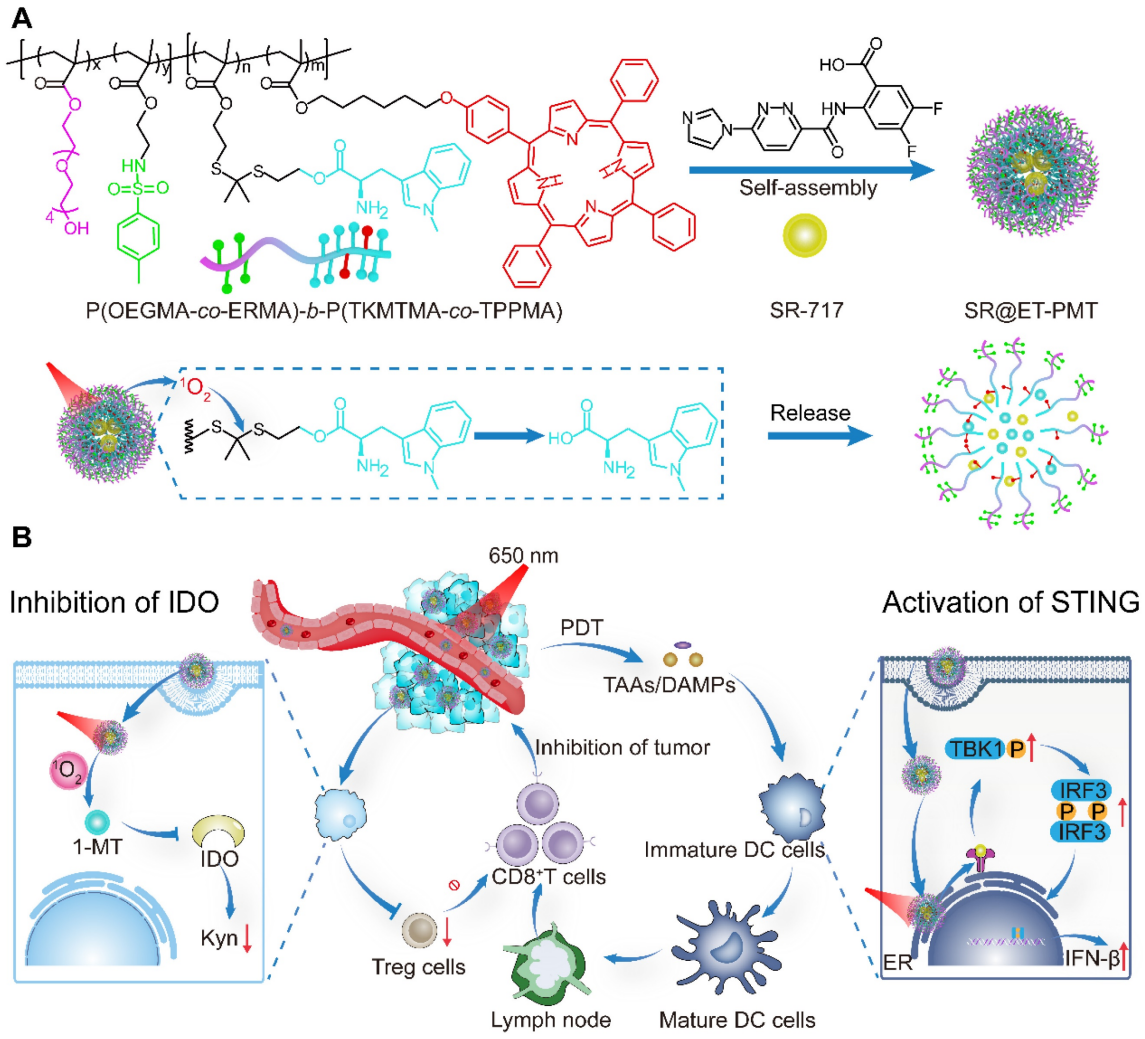
(**A**) Construction of SR@ET-PMT nanoparticles and the mechanism of photoactivated dissociation and drug release. (**B**) SR@ET-PMT boosts the synergistic antitumor immunity effect of PDT, STING agonists, and IDO inhibitor. SR@ET-PMT stimulates STING-mediated immunity through the release of SR-717 and enhances the secretion of CD8^+^ T cells. SR@ET-PMT releases 1-MT under the action of ^1^O_2_ and inhibits IDO activity, thus alleviating the immunosuppressive microenvironment.

Herein, we present photoactivated polyprodrug nanoparticles of IDO inhibitor 1-MT for ER-targeting and delivery of STING agonist SR-717 (SR@ET-PMT) (**Figure 1**). The diblock copolymer polyprodrug, P(OEGMA-*co*-ERMA)-*b*-P(TKMTMA-*co*-TPPMA), was prepared by using reversible addition-fragmentation chain transfer (RAFT) polymerization with poly[oligo(ethylene glycol) methacrylate-*co*-2-((4-methylphenyl) sulfonamido)ethyl methacrylate] (P(OEGMA-*co*-ERMA)) as the hydrophilic block, and thioketal bond-linked 1-MT methacrylate and 5,10,15,20-tetraphenylporphyrin (TPP)-containing methacrylate copolymer (P(TKMTMA-*co*-TPPMA)) as the hydrophobic block. In aqueous solution, the amphiphilic block copolymer self-assembled into micelles to encapsulate SR-717 effectively through hydrophobic and electrostatic interactions. In the nanoparticles, (4-methylphenyl) sulfonamido moieties on the shells acted as the ER-targeting group for the location at ER. TPP groups in the core served as photosensitizers to produce ROS upon light irradiation, which induced PDT effect and ROS-responsive cleavage of thioketal bonds to dissociate the micelles and trigger release of 1-MT and SR-717. More importantly, the tumor immunosuppressive microenvironment was reversed by the synergistic effects of PDT, STING activation, and IDO inhibition. SR@ET-PMT finally improved antitumor immunity synergistically, which significantly inhibited the solid tumors.

## Methods

### Synthesis of monomers and polymers

The synthetic routes of 2-((4-methylphenyl) sulfonamido)ethyl methacrylate (ERMA), thioketal bond-linked methyltryptophan methacrylate (TKMTMA), 5,10,15,20-tetraphenylporphyrin (TPP)-containing methacrylate (TPPMA), and POEGMA-*b*-P(TKMTMA-*co*-TPPMA) were shown in Supplementary Material. ^1^H NMR spectra of the monomers and the polymers were shown in **Figure S2-S11**.

Synthesis of P(OEGMA-*co*-ERMA). Oligo(ethylene glycol) methacrylate (OEGMA) (1000 mg, 3.33 mmol, 40 equiv.), 2-cyano-2-propyl benzodithioate (18.4 mg, 0.083 mmol, 1 equiv.), ERMA (354.2 mg, 1.25 mmol, 15 equiv.), and AIBN (1.9 mg, 0.012 mmol, 0.14 equiv.) were charged into a Schlenk flask containing 1,4-dioxane (5 mL). The above solution was degassed by three freeze-pump-thaw cycles and sealed under vacuum. The flask was then placed into 70 ^o^C oil bath. The polymerization lasted 24 h, then the flask was quenched into liquid nitrogen to terminate the polymerization. The mixture was precipitated into an excess of diethyl ether to generate pink residues, and the residues were collected by centrifugation. The dissolution and precipitation cycles were repeated for three times. The final product was dried in a vacuum oven overnight at room temperature, affording the resultant P(OEGMA-*co*-ERMA) as a pink sticky solid (900 mg, yield: 66.7%, *M*_n_ = 7000 Da *M*_w_/*M*_n_ = 1.04). The degrees of polymerization (DPs) of OEGMA and ERMA were determined to be 18 and 7, respectively, by using ^1^H NMR analysis (**Figure S12**).

Synthesis of P(OEGMA-*co*-ERMA)-*b*-P(TKMTMA-*co*-TPPMA). P(OEGMA-*co*-ERMA) (176 mg, 0.024 mmol, 1 equiv.), TKMTMA (336.7 mg, 0.60 mmol, 27 equiv.), TPPMA (95.3 mg, 0.12 mmol, 5 equiv.), and AIBN (0.55 mg, 0.0033 mmol, 0.14 equiv.) were charged into a Schlenk flask containing 1,4-dioxane (3 mL). The above solution was degassed by three freeze-pump-thaw cycles and sealed under vacuum. The flask was then placed into 70 ^o^C oil bath. The polymerization lasted 24 h, and then the flask was quenched into liquid nitrogen to terminate the polymerization. TFA (5 mL) was added to the mixture. The mixture was stirred for 12 h and then dialyzed against DMF and DI water to remove small molecules (MWCO, 5000 Da). The dialyzed solution was lyophilized to obtain the desired product P(OEGMA-*co*-ERMA)-*b*-P(TKMTMA-*co*-TPPMA) (300 mg, yield: 56.8%, *M*_n_ = 22000 Da, *M*_w_/*M*_n_ = 1.23). DPs of OEGMA, ERMA, TKMTMA and TPPMA were determined to be 18, 7, 28, and 3, respectively, according to ^1^H NMR analysis (**Figure S13**).

### Preparation of SR-717 delivery nanoparticles

SR@ET-PMT nanoparticles were prepared by nanoprecipitation method. Briefly, the tetrahydrofuran (THF) solution (1 mL) containing P(OEGMA-*co*-ERMA)-*b*-P(TKMTMA-*co*-TPPMA) (5 mg) and SR-717 (0.5 mg) was quickly added to phosphate buffered saline (PBS, pH 7.4, 5 mL) under rapidly stirring. THF was removed via dialysis (MWCO, 5000 Da) against PBS. The loading efficiency was calculated by the following equation: loading efficiency (%) = (weight of loaded SR-717)/(weight of SR-717 in feed) × 100%. According to similar procedures, SR@PMT without ER targeting groups and SR-717-free nanoparticles (ET-PMT and PMT) were prepared.

### Drug release profiles

The dialysis diffusion method was used to test the release of 1-MT. PMT or ET-PMT (1 mL) were loaded into dialysis bags (MWCO, 5000 Da) and placed in PBS (8 mL) with H_2_O_2_ (100 mM) at 37 °C. After taking the medium (1 mL) at different time points, the same volume of medium was replenished. The 1-MT content was measured by detecting the absorbance at a wavelength of 290 nm.

PMT or ET-PMT solutions were irradiated for 10 min by the red LED light (640-660 nm) at an intensity of 200 mW/cm^2^. The PMT or ET-PMT solutions with or without laser irradiation and pure 1-MT solution were analyzed by high performance liquid chromatography (HPLC). The conditions for HPLC assay were shown as below: methanol/H_2_O (v/v) = 82:18; flow rate = 1 mg/mL; detection wavelength = 290 nm.

The dialysis diffusion method was also used to test the release of SR-717. First, the light (640-660 nm, 200 mW/cm^2^) irradiated the solution of SR@PMT or SR@ET-PMT for 10 min. Then, SR@PMT or SR@ET-PMT (1 mL) were loaded into dialysis bags (MWCO, 5000 Da) and placed in PBS (8 mL), and incubated at 37 °C. After taking the medium (1 mL) at different time points, the same volume of medium was replenished. The SR-717 content was measured by HPLC at a wavelength of 290 nm.

### ER targeting

4T1 cells were incubated in confocal dishes at a density of 1 × 10^6^ cells per dish and cultured overnight. The cells were incubated with SR@PMT or SR@ET-PMT (TPP concentration, 15 μg/mL) for 6 h. Next, the cell culture medium was removed and washed. The cells were stained with ER-Tracker Green for 20 min. The cells were observed by CLSM. The colocalization levels were quantified by Pearson's coefficients by using the Image J software.

### *In vitro* Kyn content measurement

4T1 cells were incubated in 96-well plates at a density of 1 × 10^4^ cells/well and cultured overnight. The cells were treated with IFN-γ (100 ng/mL) and the micelles (PMT, SR@PMT, ET-PMT, or SR@ET-PMT) with or without irradiation by the red LED light (640-660 nm) at an intensity of 200 mW/cm^2^ for 10 min. After incubation for 24 h, the supernatant (150 μL) was taken out followed by addition of trichloroacetic acid (75 μL, 30%) and incubation at 50 °C for 30 min. Next, an equal volume of Ehrlich's reagent (2% *p*-dimethylamino-benzaldehyde in glacial acetic acid, w/v) was added and incubated for 20 min. The absorbance of 490 nm was measured. The relative Kyn content for each group was calculated as follows: Kyn content = (absorbance in treated group/ absorbance in control group) × 100%.

### *In vivo* antitumor efficacy and immunity activation

When the tumor volume reached about 100 mm^3^, the mice (n = 30) were randomly divided into six groups, PBS (G1), SR/MT (G2), SR@PMT (G3), SR@ET-PMT (G4), SR@PMT+L (G5) or SR@ET-PMT+L (G6). The mice were administered with different groups at an equivalent TPP dose of 15 mg/kg, respectively. After 24 h post injection, the tumors were irradiated with the red LED light (640-660 nm) at an intensity of 200 mW/cm^2^ for 10 min. Tumor volumes and mice body weights were measured every two days. The tumor volume and tumor growth inhibition value (TGI) were calculated by the following equation: volume = (tumor length) × (tumor width)^2^/2, TGI = [1-(V/V_0_)_treatment group_/(V/V_0_)_PBS group_] × 100%. V: tumor volume on day 15. V_0_: tumor volume on day 1.

For immunity activation evaluation, after 7 days of treatment, lymph nodes, blood, and tumors were collected from the mice (n = 3). Subsequently, the cells in lymph nodes and tumors were co-stained by fluorescence-labeled antibody (CD11c, CD80, CD86, CD8, and CD3) for flow cytometry analysis to measure the mature dendritic cells (DCs) and CD8^+^ T cells. In addition, the content of proinflammatory cytokines, tumor necrosis factor-α (TNF-α), interferon β (IFN-β), and interleukin 6 (IL-6) in serum and tumors were tested by enzyme linked immunosorbent assay (ELISA) according to the manufacture's protocol. Next, the tumor tissue homogenate was added with 30% trichloroacetic acid and incubated at 50 ^o^C for 30 min. Next, an equal volume of Ehrlich's reagent (2% p-dimethylamino-benzaldehyde in glacial acetic acid, w/v) was added and incubated for 20 min. The absorbance of 490 nm was measured.

### Statistical analysis

The results in all experiments were expressed as mean ± s.d.. Statistical calculation of experimental data was performed using the One-way ANOVA statistical analysis. The data were classified according to the *p* values and denoted by (*) for *p* < 0.05, (**) for *p* < 0.01, and (***) for *p* < 0.001.

## Results and discussion

### Synthesis and self-assembly of the amphiphilic polyprodrugs

The application of polyprodrugs in targeted drug delivery has garnered significant attention due to their advantages including the prevention of premature drug leakage, spatiotemporal control of drug release, a fixed drug loading content, and versatile assembly morphologies 55-59. To enhance the effect of STING immunity, we designed a polyprodrug that could target the ER and regulate the immunosuppressive environment. The amphiphilic diblock copolymer polyprodrug that can release 1-MT in response to ROS were designed to self-assemble into micelles for delivery of STING agonist SR-717. We first synthesized the monomers including ERMA with 4-methylphenyl sulfonamido as the ER-targeting groups 60, 61, TKMTMA with thioketal bond-linked IDO inhibitor 1-MT, and TPPMA with TPP photosensitizers 54, 62, 63. The synthetic routes were described in **Figure S1**. All the monomers were characterized sufficiently by ^1^H NMR analysis (**Figure S2-S9**).

**Figure 2:**
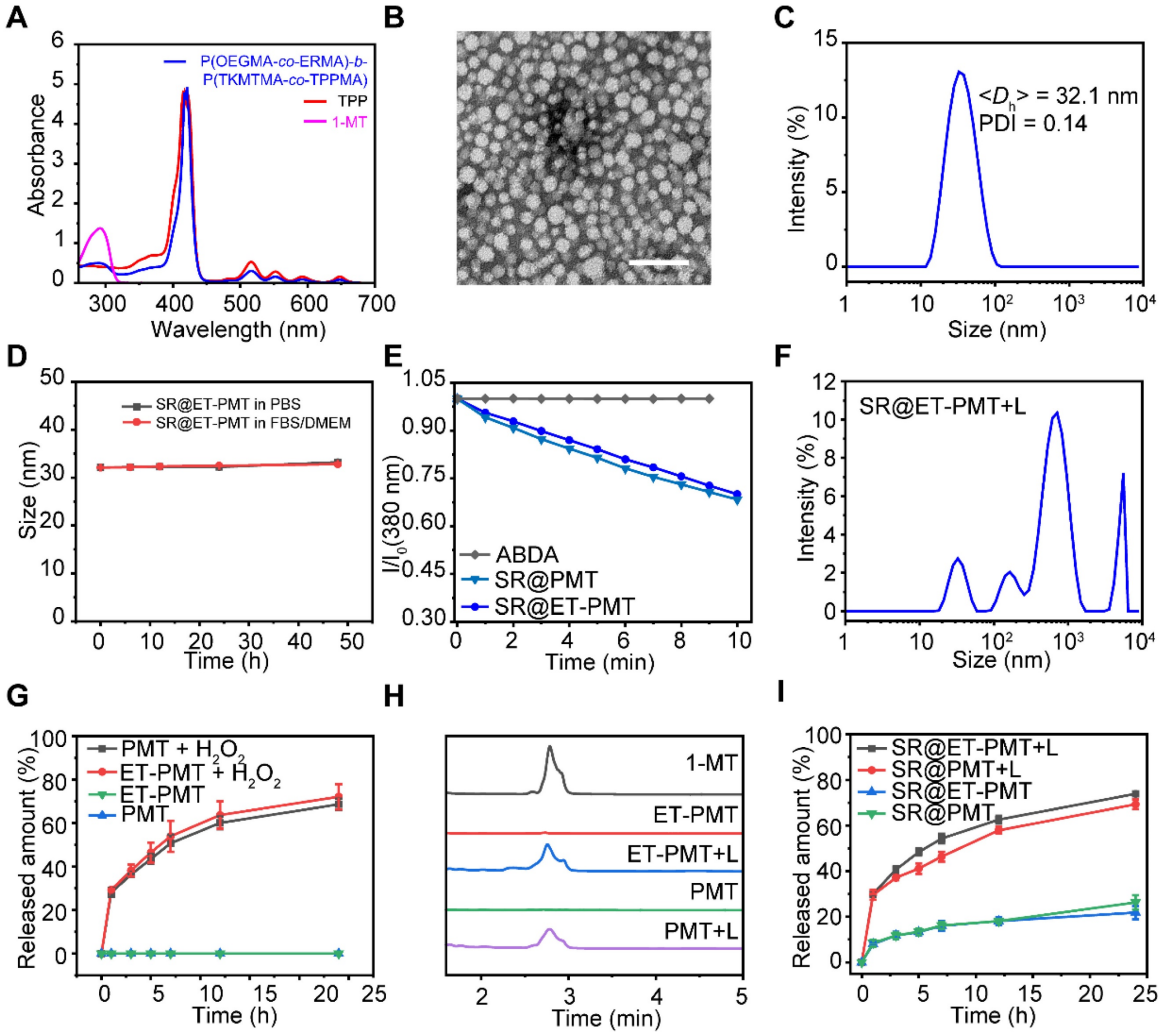
(**A**) UV-vis absorbance spectra of 1-MT, TPP and P(OEGMA-c*o*-ERMA)-*b*-P(TKMTMA-*co*-TPPMA) in chloroform. (**B**) TEM images of SR@ET-PMT. Scale bar is 100 nm. (**C**) Size distribution of SR@ET-PMT. (**D**) Time-dependent size change of SR@ET-PMT in PBS or DMEM with 10% FBS. (**E**) Time-dependent absorbance at 380 nm of ABDA in the presence of SR@PMT and SR@ET-PMT. I_0_: absorbance at 0 min, I: absorbance at different time points from 1 to 10 min. (**F**) Size distribution of SR@ET-PMT after 10 min of light irradiation (640-660 nm, 200 mW/cm^2^). (**G**) 1-MT release profiles from ET-PMT and PMT in the absence or presence of H_2_O_2_ (100 mM). (**H**) HPLC curves of 1-MT, ET-PMT, PMT with or without light irradiation for 10 min. (**I**) SR-717 release profiles from SR@ET-PMT and SR@PMT in the absence or presence of light. Mean ± s. d., n = 3.

The block copolymers were prepared by the RAFT polymerization and the synthetic routes were depicted in **Figure S1**. Two diblock copolymers, POEGMA-*b*-P(TKMTMA-*co*-TPPMA) without ER-targeting groups and P(OEGMA-c*o*-ERMA)-*b*-P(TKMTMA-*co*-TPPMA) with ER-targeting groups, were produced by using a two-step RAFT polymerization. The successful synthesis of POEGMA-*b*-P(TKMTMA-*co*-TPPMA) was demonstrated by ^1^H NMR, and the DPs of OEGMA, TKMTMA and TPPMA were determined to be 20, 25, and 3 by ^1^H NMR, respectively (**Figure S10-S11**). The drug loading contents of 1-MT drug and TPP photosensitizer in POEGMA-*b*-P(TKMTMA-*co*-TPPMA) were determined to be 53.5% and 11.3%, respectively. ^1^H NMR was also used to successfully analyze P(OEGMA-c*o*-ERMA)-*b*-P(TKMTMA-*co*-TPPMA). The DPs of OEGMA, ERMA, TKMTMA and TPPMA were determined to be 18, 7, 28, and 3, respectively (**Figure S12-S13**). The drug loading capacities of MT drug and TPP photosensitizer in P(OEGMA-c*o*-ERMA)-*b*-P(TKMTMA-*co*-TPPMA) was determined to be 56.6% and 10.6%, respectively. Gel permeation chromatography (GPC) traces showed that the molecular weight distributions of the two block copolymers were relatively narrow with *M*_w_/*M*_n_ of 1.18 and 1.23 for POEGMA-*b*-P(TKMTMA-*co*-TPPMA) and P(OEGMA-c*o*-ERMA)-*b*-P(TKMTMA-*co*-TPPMA), respectively. Moreover, the elution position shift of the diblock copolymers as compared with the macroRAFT agents suggested that the well-defined block copolymers could be obtained by the controlled polymerization method (**Figure S14**). The UV-vis absorbance characterization showed that P(OEGMA-*co*-ERMA)-*b*-P(TKMTMA-*co*-TPPMA) and POEGMA-*b*-P(TKMTMA-*co*-TPPMA) had absorbance peaks at 290 and 420 nm, which were the characteristic peaks of 1-MT (290 nm) and TPP (420 nm), respectively, indicating that 1-MT and TPP were successfully incorporated into the block copolymers (**Figure 2A** and** S15**).

Next, the nanoprecipitation method was employed to prepare the micelles 55. P(OEGMA-c*o*-ERMA)-*b*-P(TKMTMA-*co*-TPPMA) and SR-717 were dissolved in THF, which was then rapidly added into stirred PBS to produce micelles designated as SR@ET-PMT. The control micelle SR@PMT was prepared by using POEGMA-*b*-P(TKMTMA-*co*-TPPMA) and SR-717. And the two micelles (PMT and ET-PMT) without SR-717 were also prepared by using the similar method. The loading efficiencies of POEGMA-*b*-P(TKMTMA-*co*-TPPMA) and P(OEGMA-*co*-ERMA)-*b*-P(TKMTMA-*co*-TPPMA) micelles for SR-717 were determined to be 18% and 20%, respectively. The relatively high SR-717 loading efficiencies could be attributed to the electrostatic interaction between the carboxyl group and the amino group, as well as the hydrophobic interaction. Subsequently, we used transmission electron microscopy (TEM) and dynamic light scattering (DLS) to further investigate the morphology and size of the micelles. TEM characterization revealed that SR@PMT and SR@ET-PMT had uniform spherical morphology with the diameters of ~19.3 and ~19.2 nm, respectively (**Figure 2B** and **S16**). DLS measurement results of SR@PMT and SR@ET-PMT showed the diameters of 35.0 ± 0.7 and 32.1 ± 0.4 nm, respectively (**Figure 2C** and **S16**). PMT and ET-PMT had slightly smaller sizes as compared to SR@PMT and SR@ET-PMT. The results indicated that SR@PMT and SR@ET-PMT had relatively suitable sizes for further *in vivo* applications.

Moreover, we further investigated the stability of micelles under different conditions (PBS and 10% fetal bovine serum (FBS)). According to DLS data, the average diameters and size distributions of SR@PMT and SR@ET-PMT basically maintained constant within 48 h (**Figure 2D** and **S17-S18**), indicating that SR@PMT and SR@ET-PMT had good stability which was conducive for subsequent applications under different conditions. Collectively, the results showed that SR@PMT and SR@ET-PMT had suitable sizes and excellent stability under different conditions, which were favorable for further applications.

### ROS generation and drug release

To verify the PDT effect of SR@ER-PMT and 1-MT release from the micelles, we firstly studied the capability to produce ROS upon exposure to light irradiation by using 9,10-anthracenediyl-bis(methylene)dimalonic acid (ABDA) as the probe. Upon exposure to irradiation with the red-light emitting diode (LED) between 640 and 660 nm, the absorbance of ABDA was evaluated to investigate the ROS generation capacity of the micelles. The absorption peaks of ABDA (356, 380, and 402 nm) could be reduced by SR@PMT and SR@ET-PMT under light irradiation as shown in **Figure S19** and** 2E**. However, the change of absorption peaks of ABDA was negligible in only ABDA group. This indicated that SR@PMT and SR@ET-PMT can generate plentiful ROS and have similar ROS production abilities under light irradiation.

ROS generation by SR@PMT and SR@ET-PMT could break the thioketal bonds and release 1-MT and SR-717, thus destroying the micellar structure. The size changes of SR@ET-PMT and drug release were further investigated under light irradiation. As shown in **Figure 2F** and **S20**, the size distributions of SR@ET-PMT and SR@PMT changed obviously under light irradiation. The nanoparticles of SR@ET-PMT and SR@PMT in TEM images collapsed under light irradiation (**Figure S21**). Some irregularly shaped aggregates can be observed. These results indicated that ROS produced by SR@ET-PMT and SR@PMT destroyed the micellar structure under light irradiation. Next, we investigated the release of 1-MT at 100 mM H_2_O_2_. **Figure 2G** illustrated that the drug release of 1-MT in PMT and ET-PMT may attain 65% and 70% within 24 h. This indicated that PMT and ET-PMT could effectively release 1-MT under the trigger of ROS. To further verify the photoactivatable release of drugs, HPLC was used to monitor the release of 1-MT (**Figure 2H**). Without light irradiation, there was no elution peak of 1-MT (2.8 min) in the ET-PMT or PMT solution. In contrast, the elution peak of 1-MT was observed in the ET-PMT or PMT solution after 10 min of light irradiation, confirming the photoactivatable release of 1-MT. Furthermore, we further investigated the release of SR-717. As shown in **Figure 2I**, the release of SR-717 in SR@ET-PMT and SR@PMT under light irradiation may attain 73% and 69%, respectively, whereas the release of SR-717 in SR@ET-PMT and SR@PMT without light irradiation only attained 21% and 26%, respectively. The results showed that SR@ET-PMT and SR@PMT could effectively release IDO inhibitor 1-MT and STING agonist SR-717 under light irradiation. This can lay a foundation for the subsequent combination therapy of PDT, IDO inhibitor, and STING agonist.

### Cellular uptake and ER targeting

**Figure 3:**
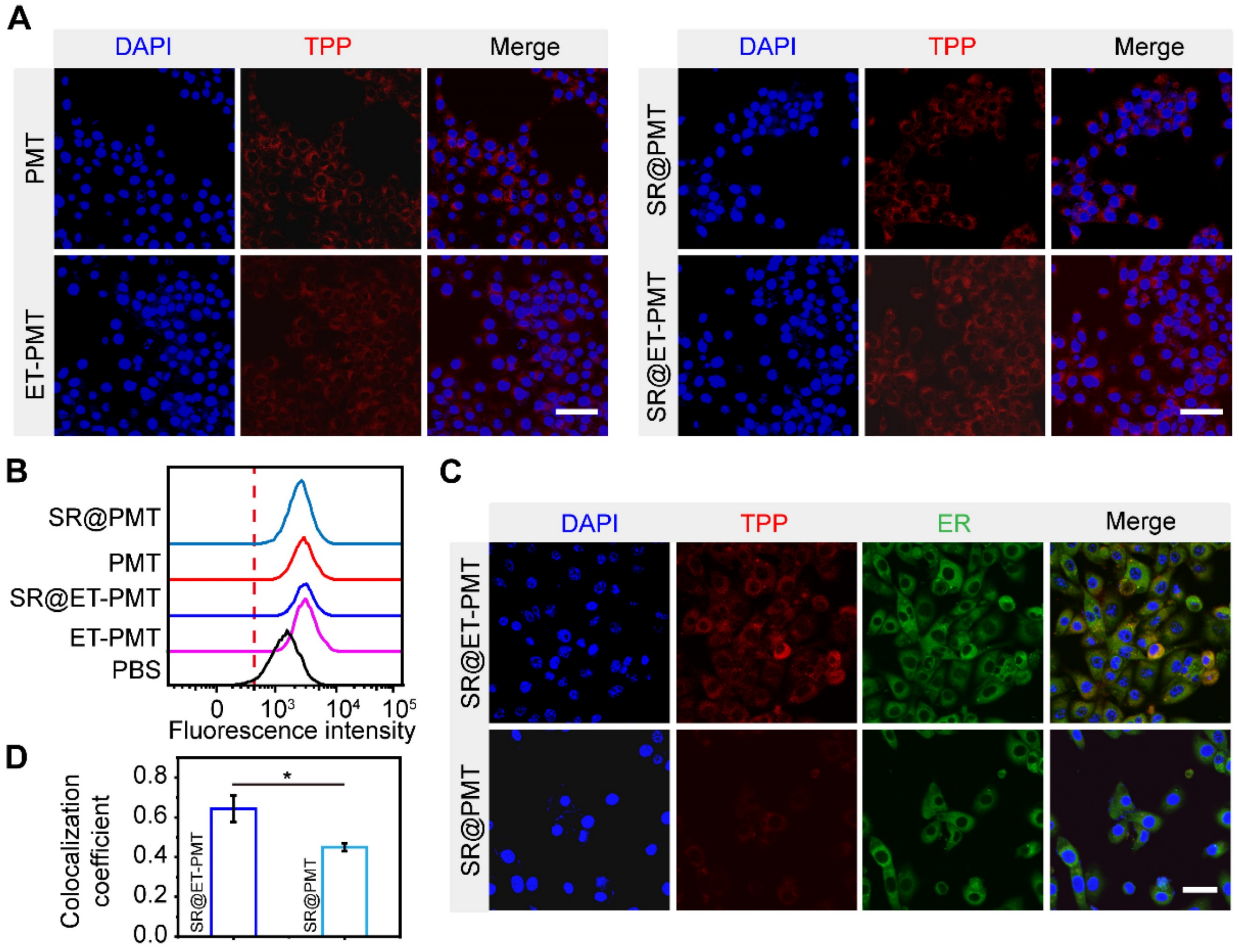
(**A**) CLSM images of 4T1 cells after incubation with PMT, SR@PMT, ET-PMT, and SR@ET-PMT for 12 h. Scale bar is 50 μm. (**B**) Flow cytometry analysis of 4T1 cells after incubation for 6 h. (**C**) CLSM images of 4T1 cells after incubation with SR@PMT or SR@ET-PMT. Scale bar is 10 μm. (**D**) Pearson's coefficients of SR@ET-PMT and SR@PMT with the ER. Mean ± s.d., n = 3.

To validate that SR@ET-PMT can target ER, we initially investigated its cellular uptake behavior by confocal laser scanning microscope (CLSM) and flow cytometry. **Figure 3A** and **S22** showed that the red fluorescence signal of TPP in 4T1 cells treated with SR@ET-PMT and SR@PMT was observed clearly at different time points indicating that SR@ET-PMT and SR@PMT could be effectively internalized by 4T1 cells. After 12 h incubation, the intracellular fluorescence intensities of both SR@ET-PMT and SR@PMT groups exhibited 7 and 5-fold increase compared to the intensity at 0 h (**Figure S23**). Furthermore, the results of flow cytometry also showed that the fluorescence intensity of SR@ET-PMT and SR@PMT gradually increased over incubation time (**Figure 3B** and **S24**). These results indicated that these micelles exhibited effective cellular internalization.

STING proteins are located on the ER of cells and STING agonists must be transported to the ER for STING activation 6, 7, 64. We further evaluated the ER-targeting capability of SR@ET-PMT. After incubation of SR@ET-PMT with 4T1 cells, ER was labeled with ER-tracker green. As shown in **Figure 3C**, SR@ET-PMT exhibited significantly greater yellow fluorescence in the ER as compared with SR@PMT group. The yellow fluorescence revealed that SR@ET-PMT micelles with the red fluorescence could overlap with ER's green fluorescence. SR@ET-PMT had a higher fluorescence overlapping ratio between the red color of TPP and the ER tracker green with the Pearson's coefficient index of ~0.64, while SR@PMT exhibited a lower Pearson's coefficient index of ~0.45. This demonstrated the superior ER targeting capability of SR@ET-PMT, which could promote the activation of STING immunity with the controlled STING agonist release.

### *In vitro* cytotoxicity

**Figure 4:**
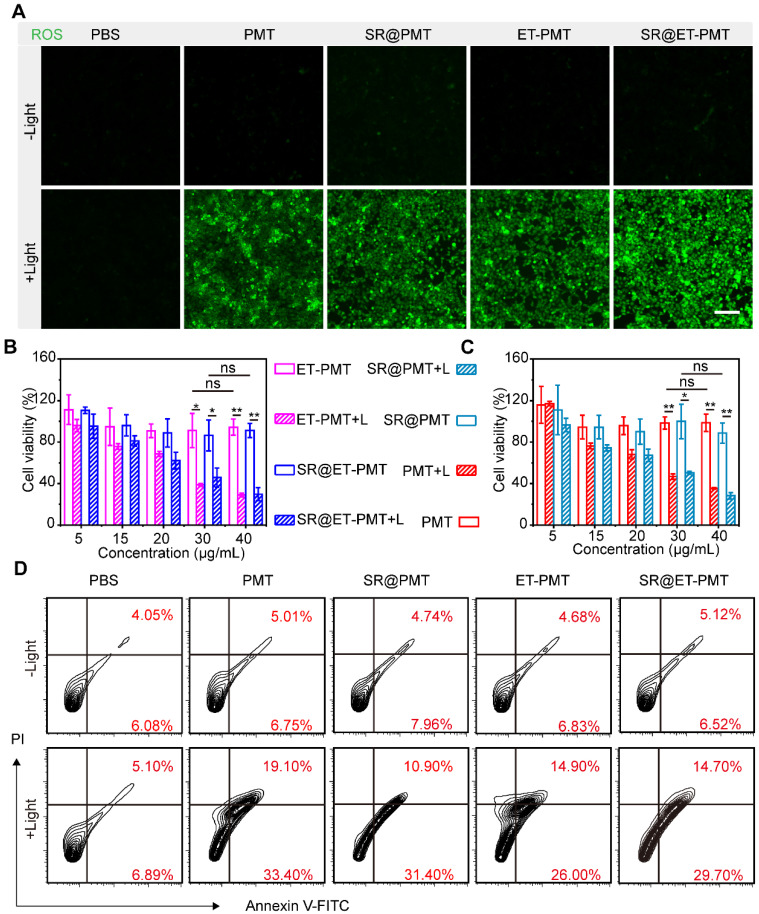
(**A**) CLSM images of ROS generation inside 4T1 cells after different treatments by using DCFH-DA as the probe. Scale bar is 100 μm. (**B**, **C**) Cell viability of 4T1 cells after 8 h incubation with ET-PMT, PMT, SR@ET-PMT, and SR@PMT (TPP-equivalent concentration) with or without light irradiation (640-660 nm, 200 mW/cm^2^, 10 min). Mean ± s.d., n = 4. (**D**) Flow cytometry analysis of 4T1 cells after staining with Annexin V-FITC and PI under different treatments.

To evaluate the ROS production by SR@ET-PMT under light irradiation, we incubated 4T1 cells in the presence of PMT, SR@PMT, ET-PMT, or SR@ET-PMT. We investigated intracellular ROS generation upon exposure to light irradiation by using 2',7'-dichlorodihydrofluorescein diacetate (DCFH-DA) as the probe. After light irradiation, significant green fluorescence was observed in 4T1 cells after treatment with SR@PMT and SR@ET-PMT (**Figure 4A**). These findings suggested that SR@PMT and SR@ET-PMT showed the strong ROS production capability upon exposure to light irradiation.

Next, *in vitro* cytotoxicity of SR@ET-PMT and SR@PMT was evaluated by cell counting kit-8 (CCK-8) assays and flow cytometry analysis (**Figure 4B** and **4C**). Without light irradiation, PMT, ET-PMT, SR@PMT, and SR@ET-PMT with different concentrations showed no significant cytotoxicity. In sharp contrast, the cell viabilities of SR@ET-PMT and SR@PMT were lower than 40% at the TPP-equivalent concentration of 40 μg/mL under light irradiation. Specifically, SR@ET-PMT+L group exhibited lower cell viability of 30% which was 3.06-fold reduction as compared with the group in the absence of light irradiation. PMT and ET-PMT had similar cytotoxicity as compared with SR@ET-PMT and SR@PMT. The IC_50_ values of SR@PMT+L group and SR@ET-PMT+L group were 27.7 μg/mL and 27 μg/mL, respectively. In addition, annexin V-FITC/PI assays were also used to evaluate cell apoptosis with different treatments (**Figure 4D**). The 4T1 cells treated with SR@PMT and SR@ET-PMT showed lower ratios of apoptotic cells without light irradiation. Conversely, the proportions of apoptotic cells of the SR@ET-PMT group (44.4%) and SR@PMT group (42.3%) were significantly higher than the PBS group (12.0%) under light irradiation. Consequently, SR@ET-PMT demonstrated superior ROS production capacity under light and effectively promoting the apoptosis of tumor cells and facilitating the release of 1-MT, which inhibited IDO activity.

### *In vitro* IDO inhibition and STING activation

**Figure 5:**
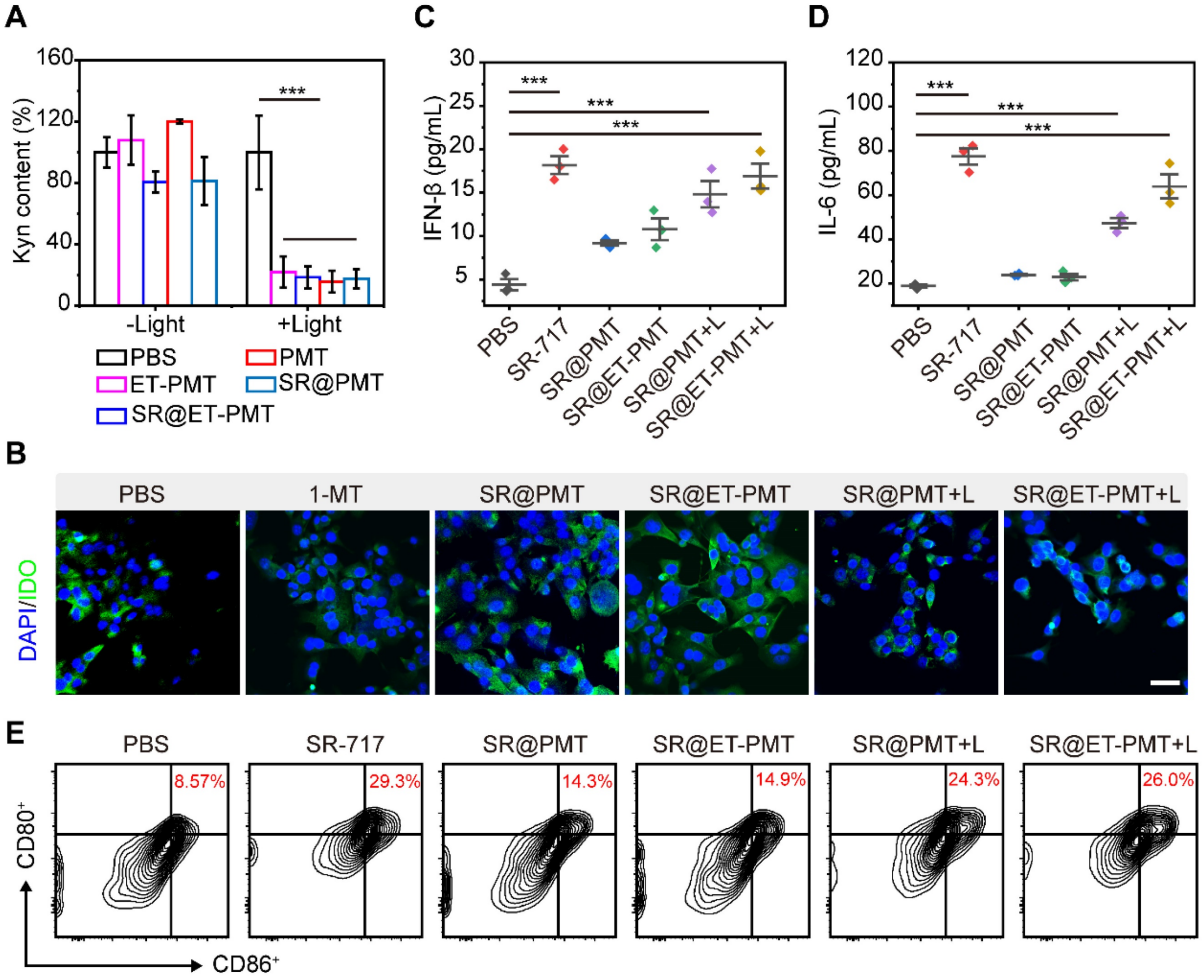
(**A**) Intracellular relative Kyn content of 4T1 cells after different treatments. (**B**) Immunofluorescence staining images of IDO (green) in 4T1 cells. The nucleus was stained with DAPI (blue). Scale bar is 50 μm. (**C-D**) Cytokine levels of IL-6, IFN-β and TNF-α in supernatants of BMDCs after different treatments. (**E**) The expression of CD80 and CD86 on BMDCs with various treatments. Mean ± s.d., n = 3.

The high concentration of ROS generated by light irradiation disrupted the thioketal linkage to release the IDO inhibitor 1-MT, which subsequently inhibited the IDO activity. To verify the inhibitory effect of the micelles on IDO activity, we studied Kyn content in the cell culture medium after different treatments. As compared with PBS group, the Kyn contents in 4T1 cells treated with SR@PMT and SR@ET-PMT were dramatically decreased under light irradiation (**Figure 5A**). According to immunofluorescence images, the expression of IDO in 4T1 cells did not change significantly after incubation with drugs and IFN-γ, indicating that the mechanism of SR@ET-PMT decreasing Kyn was the inhibition of IDO activity rather than the expression on the basis of the released 1-MT under light irradiation (**Figure 5B** and** S25**). The results were consistent with the previous reports concerning the IDO inhibitors 65-67.

To evaluate the STING activation by SR@ET-PMT under light irradiation, we incubated bone marrow-derived dendritic cells (BMDCs) in the presence of SR@PMT and SR@ET-PMT. First, we analyzed the levels of proinflammatory cytokines IFN-β and IL-6 in BMDCs. As shown in **Figure 5C** and** 5D**, the expression of IFN-β and IL-6 treated with SR@ET-PMT+L were ~3.84 times and ~3.39 times higher than that in the PBS group, respectively. The expression of IFN-β and IL-6 treated with SR@ET-PMT+L were ~1.14 times and ~1.35 times higher than that in the SR@PMT+L group, respectively. Subsequently, we investigated the DC maturation after STING activation. As shown in **Figure 5E**, the proportion of mature DCs for the SR@ET-PMT+L group (26.0%) was slightly higher as compared with SR@PMT+L group (24.3%) which were significantly than that in the PBS group (8.57%). Thus, these results indicated that SR@ET-PMT could effectively activate STING and promote DC maturation efficiently upon exposure to light irradiation.

### *In vivo* antitumor efficacy

**Figure 6:**
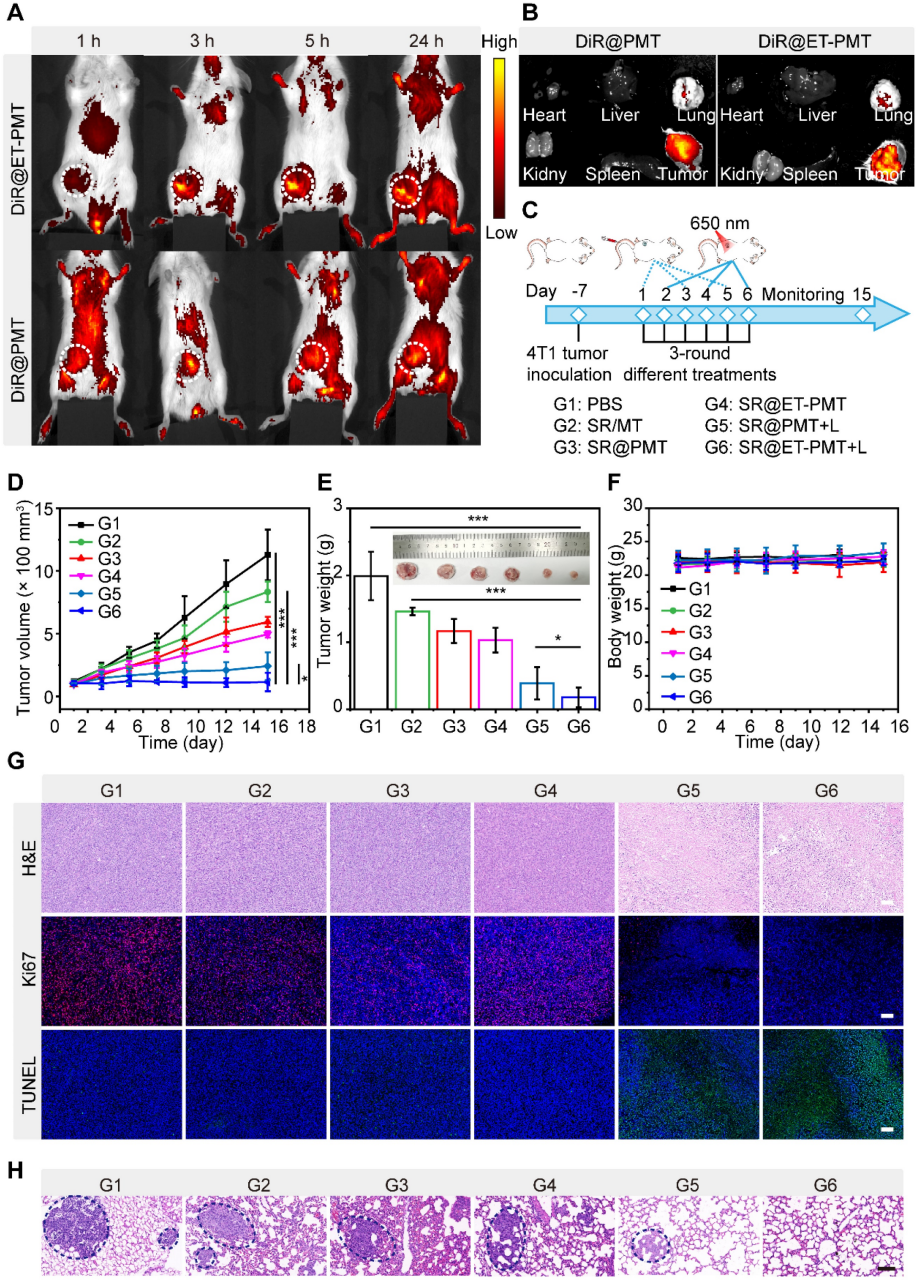
(**A**) Fluorescence images of tumor-bearing mice after intravenous injection of DiR@PMT or DiR@ET-PMT at different times. (**B**) Fluorescence images of major organs and tumors after injection of DiR@PMT or DiR@ET-PMT at 24 h. (**C**) Schematic illustration of the treatment schedule. (**D**) Growth curves of 4T1 tumors after different treatments. (**E**) Tumor weights after different treatments at day 15. (**F**) Body weights of mice after different treatments. (**G**) H&E, Ki67, and TUNEL staining images of tumor sections after different treatments. Scale bars represent 100 μm. (**H**) H&E staining of lung metastasis for different groups. Scale bars represent 100 μm. Mean ± s.d., n = 5.

Encouraged by the *in vitro* performance of the SR@ET-PMT micelles, we further evaluated the *in vivo* performance. To investigate the biodistribution and accumulation of the micelles, we prepared the 1,1'-dioctadecyl-3,3,3',3'-tetramethylindotricarbocyanine iodide (DiR)-loading micelles, DiR@PMT and DiR@ET-PMT. After intravenous injection, we observed the biodistribution of the micelles at different time points by *in vivo* imaging system (IVIS). As shown in **Figure 6A**, the fluorescence at the tumor site of mice was gradually enhanced over time, and the fluorescence reached the maximum value at 24 h after injection, indicating that the micelles could effectively accumulate at the tumor sites. Notably, the ER-targeting micelles DiR@ET-PMT showed similar biodistribution and tumor accumulation behaviors as compared with DiR@PMT. The results indicated that the blood circulation and tumor accumulation of the micelles were not affected by the introduction of ER-targeting moieties on the surface of the micelles. Meanwhile, the tumor accumulation of the two micelles were mainly determined by the nanoparticles size and stability in the physiological environment. Subsequently, the tumors and main organs including heart, liver, spleen, lung, and kidney were collected for imaging, and the strongest fluorescence in tumors could be observed for DiR@ET-PMT and DiR@PMT (**Figure 6B**).

Subsequently, to evaluate the antitumor efficacy of the combination of STING agonists and IDO inhibitors, we established 4T1 tumor models. The tumor-bearing mice were randomly assigned to six groups and treated with PBS (G1), SR/MT (G2), SR@PMT (G3), SR@ET-PMT (G4), SR@PMT+L (G5), and SR@ET-PMT+L (G6) after the tumors had grown to a size of approximately 100 mm^3^ (**Figure 6C**). During the treatment, we measured tumor volumes and body weights. After 15 days, the PBS control G1 group reached large size of ~1200 mm^3^. The tumor growth inhibition values (TGI) of the SR@ET-PMT+L group, SR@PMT+L group, SR@ET-PMT group, SR@PMT group, and SR/MT group were determined to be 88.5%, 73.3%, 45.7%, 34.0% and 17.4%, relative to the PBS group (**Figure 6D**). At the final treatment, we collected the tumors and measured the average tumor weight (**Figure 6E**). The SR@ET-PMT+L group resulted in the lowest tumor weight (0.18 g) with approximately a 11.17-fold reduction compared to the PBS group, 8.20-fold lower than SR/MT group, 6.56-fold lower than SR@PMT group, 5.80-fold lower than SR@ET-PMT group, and 2.18-fold lower than SR@PMT+L group. SR@ET-PMT with ER-targeting demonstrated superior TGI and lower average tumor weight compared to SR@PMT without ER-targeting, which was presumably attributed to the promotion of anti-tumor immunity by ER-targeting ability. Moreover, when compared to the SR@ET-PMT group, the SR@ET-PMT+L group induced a more pronounced antitumor effect, which involved PDT, light-induced release of 1-MT and SR-717. Consequently, under light irradiation, SR@ET-PMT with ER targeting could synergize PDT, STING activation, and IDO inhibition for highly efficient antitumor efficacy.

Furthermore, there was no significant change in the mice's body weights in all groups during treatment, suggesting that the nanomedicine systems showed low systemic toxicity (**Figure 6F**). **Figure 6G** displayed tumor tissues after staining with hematoxylin and eosin (H&E), Ki67, and terminal deoxynucleotidyl transferase-mediated dUTP nick end labeling (TUNEL). The H&E staining results indicated that SR@ET-PMT+L group led to significant reduction in cell density and increase in nuclear dissociation, and necrosis within the tumor tissue as compared with the other groups. TUNEL and Ki67 immunofluorescence staining images revealed significant increase in apoptotic cells (green fluorescence) in SR@ET-PMT+L group, which was accompanied by a notable decrease in tumor proliferation markers (red fluorescence), thereby further confirming the excellent antitumor efficacy. H&E staining images of lung tissues from mice treated with SR@ET-PMT+L revealed no significant metastases (**Figure 6H**). In contrast, extensive metastases were observed in lung tissues from mice in other groups. These findings revealed that SR@ET-PMT+L treatment, when administered under light, effectively inhibited tumor metastasis. Additionally, negligible damage to major organs was observed during the various treatment groups according to H&E staining images of the organs (**Figure S26**). The results showed that SR@ET-PMT had a good level of biosafety and good therapeutic effect on tumors after intravenous injection.

### *In vivo* immune activation

**Figure 7:**
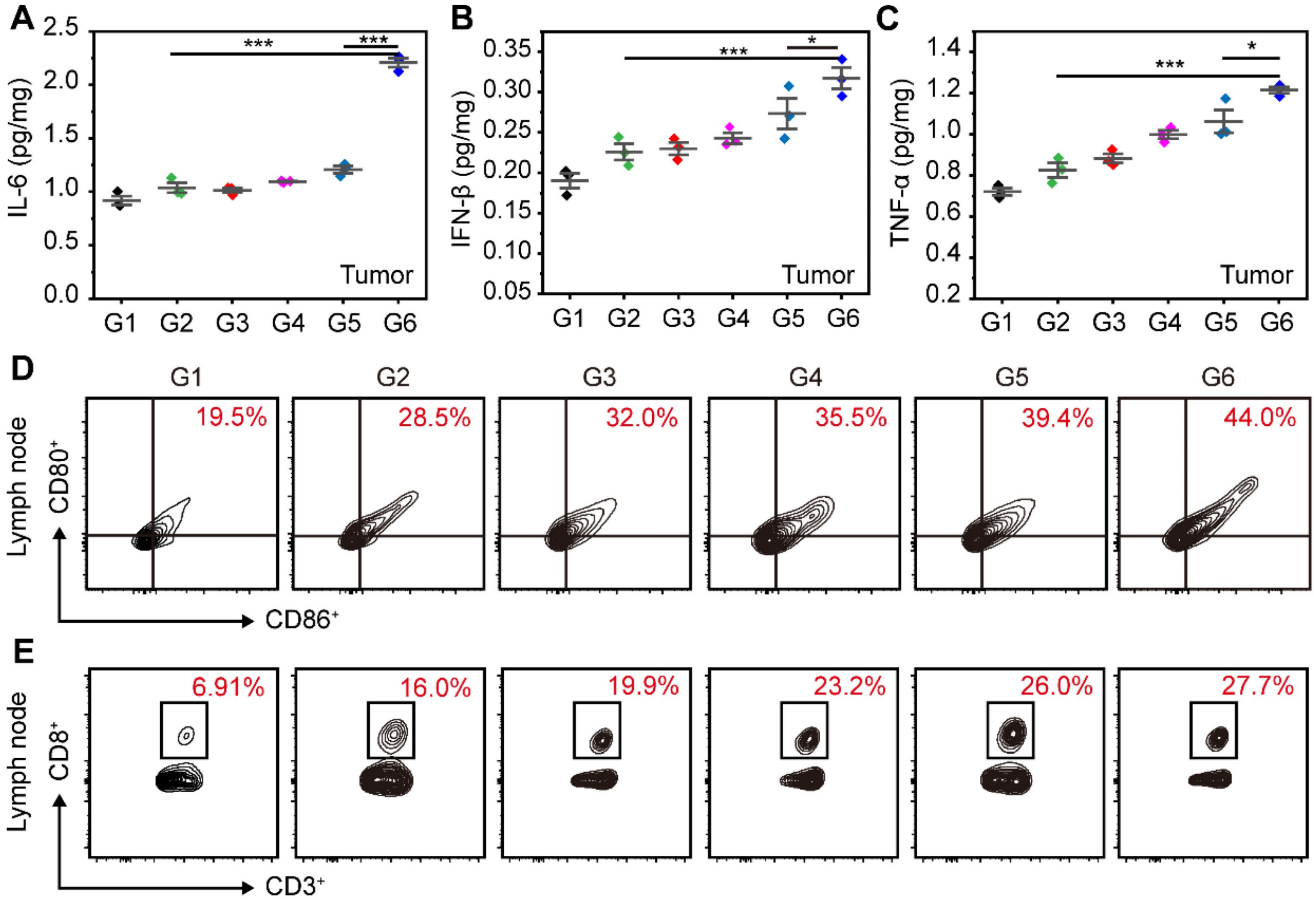
(**A**-**C**) Cytokine levels of IL-6, IFN-β and TNF-α in tumors after different treatments. (**D**-**E**) Flow cytometry analysis of mature DCs (CD80^+^ CD86^+^) and CD8^+^ T cells in Lymph nodes after different treatments. G1: PBS, G2: SR/MT, G3: SR@PMT, G4: SR@ET-PMT, G5: SR@PMT+L, G6: SR@ET-PMT+L. Mean ± s.d., n = 3.

1-MT could inhibit the activity of IDO and relieve the immunosuppressive environment, which can promote the activation of PDT and STING immunity for tumor suppression. In the ER of cells, STING agonists can interact with STING proteins to trigger the phosphorylation and dimerization of IRF3, which subsequently stimulates the expression of IFN-β. Simultaneously, the activation of STING immunity enhances the expression of pro-inflammatory cytokines, including IFN-β, TNF-α, and IL-6. The ER-targeting micelles facilitated efficient accumulation of STING agonists in ER, potentially leading to a more robust activation of STING immunity. To validate this hypothesis, we initially assessed the expression of IFN-β, TNF-α and IL-6 induced by different treatments in tumors. As shown in **Figure 7A-C**, the SR@ET-PMT+L group had the highest expression levels of various cytokines, which were 2.13-fold (IL-6), 1.40-fold (IFN-β), and 1.47-fold (TNF-α) higher as compared with those in the SR/MT group. And the levels of these cytokines in SR@ET-PMT+L group were 1.83-fold (IL-6), 1.16-fold (IFN-β), and 1.14-fold (TNF-α) higher than those in the SR@PMT+L group. These results indicated that the SR@ET-PMT+L group exhibited the most potent antitumor immune effect, potentially attributable to the role of ER targeting. This finding offered preliminary evidence for the synergistic enhancement of antitumor immunity through the combination of STING agonists and IDO inhibitors.

To explain the potential of SR@ET-PMT+L group in enhancing anti-tumor immunity, we studied the maturation of DCs and the proliferation of CD8^+^ T cells in lymph nodes. The proportion of mature DCs in lymph nodes for the SR@ET-PMT+L group (44.0%) was significantly higher as compared with SR@PMT group (39.4%), SR/MT group (28.5%), and PBS group (19.5%) (**Figure 7D**). These results indicated that SR@ET-PMT+L group was most efficient to promote DCs maturation, which can be attributed to the synergistic effect of ER targeting ability, PDT effect, as well as 1-MT and SR-717 release for immune activation. The maturation of DCs further promoted the proliferation of CD8^+^ T cells. The SR@ET-PMT+L group (27.7%) resulted in the highest proportion of CD8^+^ T cells among all groups, with approximately a 4.01-fold increase compared to the PBS group, 1.73-fold more than SR/MT group, and 1.07-fold more than SR@PMT+L group (**Figure 7E**). To sum up, SR@ET-PMT+L group had the highest proportion of mature DCs and CD8^+^ T cells, which could be attributed to synergistic effect of ER targeting, PDT effect, STING activation, and IDO inhibition.

**Figure 8:**
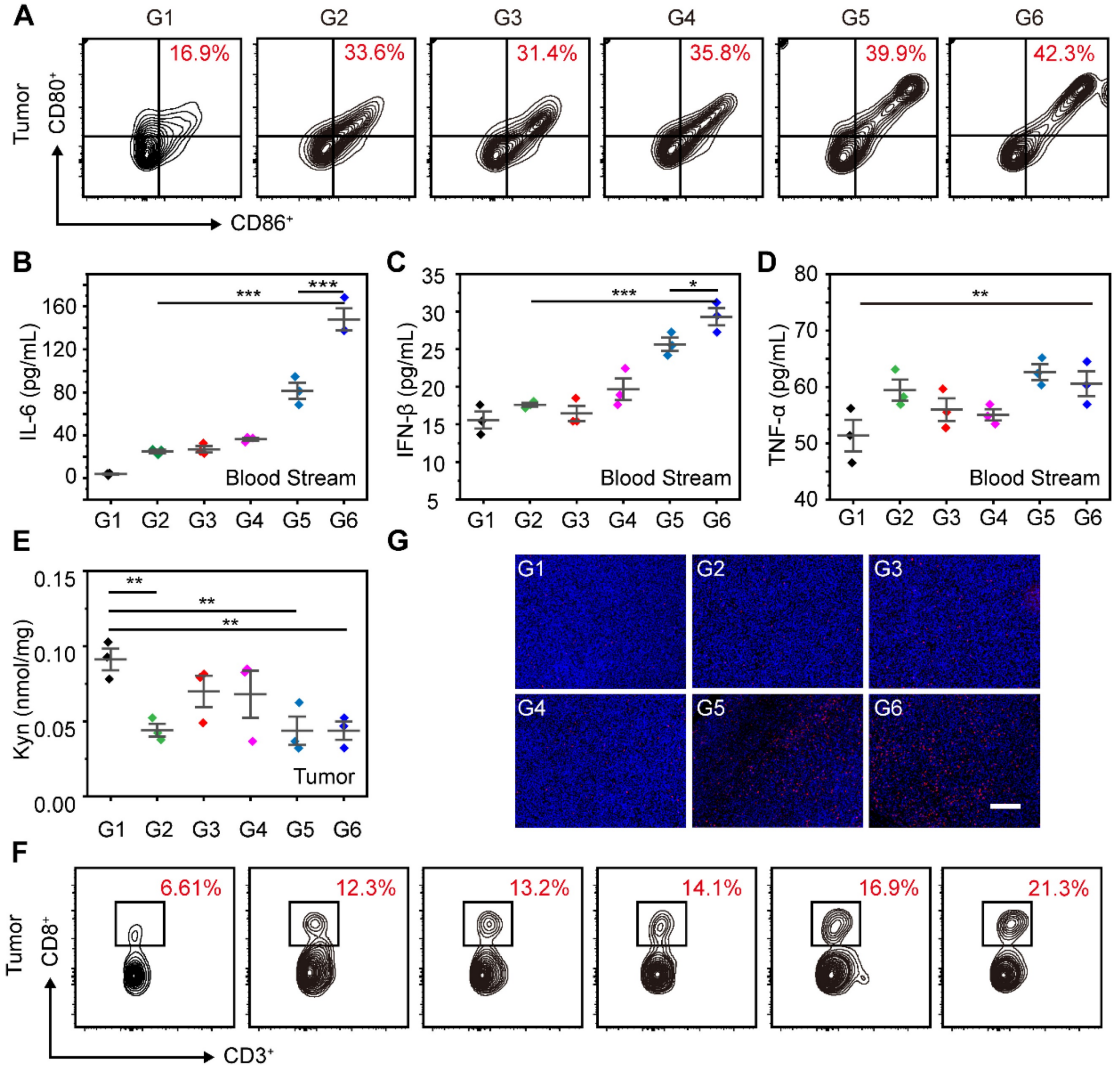
(**A**) Flow cytometry analysis of mature DCs (CD80^+^ CD86^+^) and CD8^+^ T in tumors after different treatments. (**B**-**D**) Cytokine levels of IL-6, IFN-β and TNF-α in serum after different treatments. (**E**) Kyn levels in tumors after different treatments. (**F**) Flow cytometry analysis of CD8^+^ T in tumors after different treatments. (**G**) Immunofluorescence staining images of CD8^+^ T cells (red) in tumor. The nucleus was stained with DAPI (blue). Scale bar is 200 μm. G1: PBS, G2: SR/MT, G3: SR@PMT, G4: SR@ET-PMT, G5: SR@PMT+L, G6: SR@ET-PMT+L. Mean ± s.d., n = 3.

Moreover, we further evaluated the maturation of DCs in tumors after the combination of STING agonists and IDO inhibitors. As shown in **Figure 8A**, the proportion of mature DCs in tumors for SR@ET-PMT+L group (42.3%) was significantly higher than those of SR@PMT group (39.9%), SR/MT group (33.6%), and PBS group (16.9%). To assess the systemic immune response induced by SR@ET-PMT+L group, we measured the levels of IFN-β, TNF-α, and IL-6 in the serum. After SR@ET-PMT+L group treatment, the secretion of IFN-β, TNF-α and IL-6 was significantly increased in mouse serum. The levels of these cytokines in SR@ET-PMT+L group were 1.82-fold IL-6 and 1.14-fold IFN-β expression as compared with those in SR@PMT+L group (**Figure 8B-D**). These results showed that the combination of STING agonists and IDO inhibitors can elicit systemic antitumor immune effect.

Overexpression of IDO in tumor can inhibit the proliferation of effector T cells, thereby reducing the effectiveness of immunotherapy. Next, we tested the Kyn contents after different treatments. The Kyn content was significantly reduced after SR@ET-PMT+L treatment (**Figure 8E**). This could be attributed to the release of 1-MT under light irradiation. Moreover, in tumors, the proportion of CD8^+^ T cells of the SR@ET-PMT+L group (21.3%) was higher than those of the SR@PMT group (16.9%), SR/MT group (12.3%), and PBS group (6.61%) (**Figure 8F**). Immunofluorescence staining images of CD8^+^ T cells in tumors further showed that the SR@ET-PMT+L group had the strongest red fluorescence, indicating that the SR@ET-PMT+L group secreted the largest number of CD8^+^ T cells (**Figure 8G**). The comprehensive analysis indicated that the combination therapy of STING agonists and IDO inhibitors activated robust anti-tumor immunity inside tumors and the systemic immunity was also activated effectively.

## Conclusions

In summary, we prepared the amphiphilic polyprodrugs with ER-targeting ability in the hydrophilic segment as well as IDO inhibitor 1-MT and photosensitizer TPP in the hydrophobic block. The polyprodrug could self-assemble into micelles to encapsulate SR-717 efficiently through the hydrophobic and electrostatic interactions (SR@ET-PMT). SR@ET-PMT could effectively enter 4T1 cells and target to the ER. Upon exposure to light irradiation, SR@ET-PMT produced a large amount of ROS and promoted the dissociation of micelles to release 1-MT and SR-717. After intravenous injection, SR@ET-PMT showed long blood circulation and efficient tumor accumulation. When the tumors were irradiated by the light, the release of 1-MT reduced Kyn content significantly inside tumor tissues. Moreover, SR@ET-PMT could effectively suppress tumor growth, release tumor-associated antigens and activate the STING pathway by SR-717, and promote the maturation of DCs and the secretion of CD8^+^ T cells. The effective antitumor immune response can be attributed to the synergistic effect of ER targeting, PDT effect, STING activation, and IDO inhibition. This work proposed the photoactivatable immunomodulator polyprodrugs incorporating ER targeting, STING agonists, and IDO inhibitors, which exhibited considerable potentials for boosting cancer immunotherapy outcomes and offers a pathway for the development of the combination immunotherapy.
