# Supplementary material for: Photoactivatable immunomodulator polyprodrugs for boosting synergistic antitumor immunity of STING agonists and IDO inhibitors
Source: Theranostics. 2025 Mar 8;15(9):3979–94. doi: 10.7150/thno.107774 (PMC11980649; doi:10.7150/thno.107774)
Supplement: Supplementary file 1 — Supplementary figures and tables. [file thnov15p3979s1.pdf]

Supporting Information

Photoactivatable immunomodulator polyprodrugs for boosting synergistic antitumor immunity of STING agonists and IDO inhibitors

Guopu Huang[1,3], Cheng Li[1,2], Jiale Si[3], Yufei Cao[3], Moujiang Zheng[3], Yueming Xue[3], Qinghao Zhou[3*], Zhishen Ge[1,3*], Yuanyuan Ji[1,2*]

1. Department of Geriatric General Surgery, The Second Affiliated Hospital, Xi'an Jiaotong University, Xi'an 710004, Shaanxi, China

2. Scientific Research Center and Precision Medical Institute, The Second Affiliated Hospital, Xi'an Jiaotong University, Xi'an 710004, China

3. School of Chemistry, Xi'an Key Laboratory of Sustainable Polymer Materials, Xi'an Jiaotong University, Xi'an 710049, Shaanxi, China

* Corresponding authors: Professor Qinghao Zhou, E-mail: zhouqh@xjtu.edu.cn; Professor Yuanyuan Ji, E-mail: jyy2012@xjtu.edu.cn; Professor Zhishen Ge, E-mail: gezhish@xjtu.edu.cn

1. Experimental section

Materials

1-Methyl-L-tryptophan (98%), triethylamine (TEA, 98%), acryloyl chloride (95%), 2-(7-azabenzotriazol-1-yl)-N,N,N',N'-tetramethyluronium hexafluorophosphate (HATU, 98%), 2-cyano-2-propyl benzodithioate (97%), N,N'-dicyclohexylcarbodiimide (DCC, 98%), 2-mercaptoethanol (99%), *p*-toluenesulfonic acid monohydrate (PTSA, 98%), methacryloyl chloride (97%), di-tert-butyl decarbonate (Boc_2O, 98%), pyrrole (99%), benzaldehyde (99%), p-hydroxybenzaldehyde (99%), 6-chloro-1-hexanol (98%), p-toluenesulfonyl chloride (99%), 2-aminoethyl methacrylate hydrochloride (AEMA, 90%), 2,2′-azobis(2-methylpropionitrile) (AIBN, 98%), polyethylene glycol monomethacrylate (OEGMA), 2,2'-Azobis(2-methylpropionitrile) (AIBN) was recrystallized twice from methanol. Fetal bovine serum (FBS), Dulbecco's modified Eagle's medium (DMEM), phosphate buffer saline (PBS, pH 7.4), and trypsin were purchased from Gibco Company (USA) and used as received. 2′,7′-Dichlorofluorescin diacetate (DCFH-DA, reactive oxygen species (ROS) probe), Cell Counting Kit-8, hematoxylin and eosin (H&E) staining kit, the terminal deoxynucleotidyl transferase-mediated dUTP nick end-labeling (TUNEL) cell apoptosis detection kit, and 2-(4-Amidinophenyl)-6-indolecarbamidine dihydrochloride (DAPI) staining solution were purchased from Beyotime Institute of Biotechnology (Shanghai, China). Enzyme linked immunosorbent assay (ELISA) kits of interleukin-6 (IL-6), interferon-β (IFN-β)

and TNF-α, antibodies against CD11c, CD80, CD86, CD3, CD8 for flow cytometry were all obtained from Southern Biotechnology Associates, Inc. All others commercially available solvents and reagents were purchased from Sinopharm Chemical Reagent Co. Ltd. and Energy Chemical (Shanghai, China) and used as received. Female BALB/c nude mice at the age of 5-6 weeks were purchased from GemPharmatec Co. Ltd. All the animal experiments were performed in compliance with the guidelines of the Institutional Animal Care and Use Committee (IACUC) established by the Health Science Center of Xi'an Jiaotong University. The case number of ethics is 2021-1080. 4T1 cells was obtained from Cell Bank of Chinese Academy of Sciences (Shanghai, China). And 4T1 cells were cultured in DMEM with 10% FBS at 37 °C under 5% CO_2.

Synthesis of ERMA

AEMA (2 g, 12.10 mmol, 1 equiv.) and TEA (3.66 g, 36.20 mmol, 3 equiv.) were added to a round-bottom flask containing DCM (100 mL), then cooled in ice bath and ventilated with nitrogen for 15 min. Subsequently, 4-toluenesulfonyl chloride (2.78 g, 14.47 mmol, 1.2 equiv.) in 20 mL DCM was added dropwise in the system under the protection of ice bath and nitrogen, and the reaction was continued for 24 h magnetically. The product 2-((4-methylphenyl) sulfonamide) ethyl methacrylate (ERMA, yellowish solid, 2.68 g, 78% yield) was separated by column chromatography (DCM). The chemical structure of ERMA was characterized and shown in Figure S2. ^{1}H NMR (400

MHz, $CDCl_3$, ppm): 7.74 (d, J = 8.4 Hz, 2H), 7.30 (d, J = 8.1 Hz, 2H), 6.04 (s, 1H), 5.56 (t, J = 1.6 Hz, 1H), 4.85 (t, J = 6.2 Hz, 1H), 4.18 – 4.12 (m, 2H), 3.28 – 3.22 (m, 2H), 2.41 (s, 3H), 1.88 (t, J = 1.3 Hz, 3H).

Synthesis of TKMTMA

Synthesis of TKOH. 2-mercaptoethanol (13.43 g, 171.89 mmol, 3 equiv.), acetone (3.32 g, 57.19 mmol, 1 equiv.), and p-toluenesulfonic acid monohydrate (0.33 g, 1.73 mmol, 0.03 equiv.) were dissolved in DCM (30 mL) and the mixture was stirred for another 4 h at 40 °C. Evaporation in vacuum and purification by flash column chromatography (DCM/EA = 5/1) gave the product as colorless oil (5.00 g, 45% yield). The chemical structure of TKOH was characterized and shown in Figure S3. ^{1}H NMR (400 MHz, D_2O, ppm): 3.65 (t, J = 6.3 Hz, 4H), 2.73 (t, J = 6.4 Hz, 4H), 1.51 (s, 6H).

Synthesis of TKOHMA. In a round bottom flask, TKOH (3.66 g, 18.64 mmol, 1 equiv.) and TEA (2.83 g, 27.96 mmol, 1.5 equiv.) were dissolved in of anhydrous CH_2Cl_2 (60 mL). Acryloyl chloride (1.95 g, 18.64 mmol, 1 equiv.) in dry CH_2Cl_2 (20 mL) was added dropwise over the course of 30 min at 0 ºC. The reaction was then warmed slowly to room temperature and stirred overnight at room temperature. The crude solid was purified on a silica column (DCM/EA = 10/1) to obtain the product (1.91 g, 39% yield). The chemical structure of TKOHMA was characterized and shown in Figure S4. ^{1}H NMR (400 MHz, $CDCl_3$, ppm): 6.12 (q, J = 1.1 Hz, 1H), 5.57 (t, J = 1.6 Hz, 1H), 4.30 (t, J = 7.0 Hz, 2H), 3.78 (t, J = 6.1 Hz, 2H), 2.92 (t, J = 7.0 Hz, 2H), 2.84 (t, J = 6.1 Hz,

2H), 1.93 (t, J = 1.3 Hz, 3H), 1.62 (s, 6H).

Synthesis of Boc-1MT. 1-Methyltryptophan (1.00 g, 4.58 mmol, 1 equiv.), $NaHCO_3$ (1.15 g, 13.74 mmol, 3 equiv.), and di(tert-butyl) dicarbonate (1.20 g, 5.50 mmol, 1.2 equiv.) were dissolved in an equal volume of THF/H_2O (1/1, 20 mL) and the mixture was stirred at 0 °C for 10 min and then at room temperature for 24 h. THF was evaporated and the aqueous layer was acidified with 1 M HCl (10 mL) to pH 1 and then extracted with ethyl acetate (3×15 mL). The ethyl acetate was evaporated to leave a pure pale white solid (1.46 g, 98% yield). The chemical structure of Boc-1MT was characterized and shown in Figure S5. ^{1}H NMR (400 MHz, $CDCl_3$, ppm): 7.61 (d, J = 8.0 Hz, 1H), 7.31 (d, J = 8.2 Hz, 1H), 7.25 (t, J = 7.5 Hz, 1H), 7.13 (t, J = 7.5 Hz, 1H), 6.93 (s, 1H), 5.05 (s, 1H), 4.66 (s, 1H), 3.76 (s, 3H), 3.40 – 3.28 (m, 2H), 1.45 (s, 9H).

Synthesis of TKMTMA. DCC (1.84 g, 8.92 mmol, 2 equiv.) and DMAP (0.54 g, 4.46 mmol, 1 equiv.) were added into the solution of TKOHMA (1.42 g, 5.35 mmol, 1.2 equiv.) in 50 mL dichloromethane. Then the solution of Boc-1MT (1.42 g, 4.46 mmol, 1 equiv.） in dichloromethane (50 mL) was dropwisely added into the reaction solution. After stirring at room temperature for 24 h, the solvent was evaporated and the residue was purified by column chromatography (PE/EA = 5/1) to get product as a colorless oil (2.52 g, 84% yield). The chemical structure of TKMTMA was characterized and shown in Figure S6. ^{1}H NMR (400 MHz, $CDCl_3$, ppm): 7.55 (d, J = 7.9 Hz, 1H), 7.29 (s, 1H), 7.21 (t, J = 7.6 Hz, 1H), 7.10 (s, 1H), 6.90 (s, 1H), 6.12 (s, 1H), 5.60 – 5.56 (m, 1H), 5.06 (d, J = 8.2 Hz, 1H), 4.62 (s, 1H), 4.29 (t, J = 7.0 Hz, 2H), 4.23 – 4.17 (m, 2H),

3.75 (s, 3H), 3.30 – 3.22 (m, 2H), 2.88 (s, 2H), 2.77 (s, 2H), 1.94 (s, 3H), 1.59 (s, 6H), 1.43 (s, 9H).

Synthesis of TPPMA

Synthesis of TPP-OH. Benzaldehyde (5.47 mL, 54 mmol, 3 equiv.) and p-hydroxybenzaldehyde (2.20 g, 18 mmol, 1 equiv.) were dissolved in propionic acid (240 mL). This solution was placed in a three-necked flask with a reflux condenser and a magnetic stirring bar, and then was heated to 135 °C for 4 h. Then freshly distilled pyrrole (5 mL, 72 mmol, 4 equiv.) was added dropwise to the solution under argon. The reaction mixture was heated to reflux for another 4 h and then cooled to room temperature. Then about half volume of the reaction mixture was removed and methanol (200 mL) was added to the concentrated solution. This solution was stored overnight in a refrigerator, and then the purple precipitation was filtered off and washed with cold methanol. The crude product was purified on silica gel column (DCM/MeOH = 60/1) to obtain the purple solid (0.50 g, 5% yield). The chemical structure of TPP-OH was characterized and shown in Figure S7. ^{1}H NMR (400 MHz, $CDCl_3$, ppm): 8.89 – 8.82 (m, 8H), 8.21 (dd, J = 7.6, 1.8 Hz, 6H), 8.07 (d, J = 8.4 Hz, 2H), 7.75 (d, J = 7.5 Hz, 9H), 7.19 (d, J = 8.4 Hz, 2H), -2.77 (s, 2H).

Synthesis of TPP6C-OH. TPP-OH (0.52 g, 0.82 mmol, 1 equiv.), 6-chloro-1-hexanol (0.13 g, 0.95 mmol, 1.16 equiv.) and potassium carbonate (0.12 g, 0.87 mmol, 1.06 equiv.) were dissolved in 100 mL DMF. The mixture solution was refluxed for 12 h and washed with water three times. The product was extracted with dichloromethane, and

dried with anhydrous $MgSO_4$. The crude product was purified on silica gel column (DCM/MeOH = 120/1) to obtain the purple solid (0.40 g, 67% yield). The chemical structure of TPP6C-OH was characterized and shown in Figure S8. ^{1}H NMR (400 MHz, $CDCl_3$, ppm): 8.91 – 8.80 (m, 8H), 8.25 – 8.18 (m, 6H), 8.11 (d, J = 8.5 Hz, 2H), 7.79 – 7.71 (m, 9H), 7.28 (s, 2H), 4.25 (s, 2H), 3.74 (d, J = 4.4 Hz, 2H), 2.00 (dd, J = 8.3, 6.5 Hz, 2H), 1.76 – 1.56 (m, 6H), -2.77 (s, 2H).

Synthesis of TPPMA. Under a nitrogen atmosphere, the precursor TPP6C-OH (0.40 g, 0.55 mmol, 1 equiv.) and trimethylamine (0.066 g, 0.66 mmol, 1.2 equiv.) were dissolved in 50 ml of DCM and chilled to 0 ℃. And then methacryloyl chloride (0.069 g, 0.66 mmol, 1.2 equiv.) was added drop wise to the solution while stirring. The mixture was allowed to warm to room temperature while stirring overnight. The crude product was further purified by column chromatography (DCM) to obtain the purple solid (0.17 g, 39% yield). The chemical structure of TPPMA was characterized and shown in Figure S9. ^{1}H NMR (400 MHz, $CDCl_3$, ppm): 8.93 – 8.74 (m, 9H), 8.25 – 8.20 (m, 6H), 8.11 (d, J = 8.6 Hz, 2H), 7.79 – 7.71 (m, 9H), 7.26 (d, J = 8.5 Hz, 2H), 6.15 (dd, J = 1.8, 1.0 Hz, 1H), 5.58 (t, J = 1.7 Hz, 1H), 4.24 (td, J = 6.5, 3.8 Hz, 4H), 2.05 – 1.95 (m, 5H), 1.86 – 1.76 (m, 2H), 1.74 – 1.65 (m, 2H), 1.63 – 1.55 (m, 2H), -2.76 (s, 2H).

Synthesis of POEGMA-*b*-P(TKMTMA-*co*-TPPMA)

Synthesis of POEGMA. The synthesis of POEGMA followed the reversible addition-fragmentation chain transfer (RAFT) polymerization technique. Briefly, OEGMA (3.00

g, 10.00 mmol, 40 equiv.), 2-cyano-2-propyl benzodithioate (55.3 mg, 0.25 mmol, 1 equiv.), and AIBN (5.7 mg, 0.035 mmol, 0.14 equiv.) were charged into a Schlenk flask containing 1,4-dioxane (5 mL). The above solution was degassed by three freeze–pump–thaw cycles and sealed under vacuum. The flask was then placed into 70 °C oil bath. The polymerization lasted 24 h, then the flask was quenched into liquid nitrogen to terminate the polymerization. The mixture was precipitated into an excess of diethyl ether to generate pink residues, the residues were collected by centrifugation. And the dissolution and precipitation cycles were repeated for three times. The final product was dried in a vacuum oven overnight at room temperature, affording the resultant POEGMA as a pink sticky solid (2.2 g, yield: 73.3%, M_n = 6000 Da, M_w/M_n = 1.12). The degree of polymerization of OEGMA determined to be ~20 based on the ^{1}H NMR analysis (Figure S10).

Synthesis of POEGMA-*b*-P(TKMTMA-*co*-TPPMA). P(OEGMA) (179 mg, 0.024 mmol, 1 equiv.), TKMTMA (364.7 mg, 0.65 mmol, 27 equiv.), TPPMA (95.5 mg, 0.12 mmol, 5 equiv.), and AIBN (0.55 mg, 0.0033 mmol, 0.14 equiv.) were charged into a Schlenk flask containing 1,4-dioxane (5 mL). The above solution was degassed by three freeze–pump–thaw cycles and sealed under vacuum. The flask was then placed into 70 °C oil bath. The polymerization lasted 24 h, then the flask was quenched into liquid nitrogen to terminate the polymerization. TFA (5 mL) was added to the mixture. And the mixture was continued for 12 h magnetically. Then the mixture was dialyzed against DMF and DI water to remove small molecules (MWCO, 5000 Da). The dialyzed

solution was lyophilized to obtain the desired product POEGMA-*b*-P(TKMTMA-*co*-TPPMA) (230 mg, yield: 47.9%, M_n = 20000 Da, M_w/M_n = 1.18). The degree of polymerization of OEGMA, TKMTMA and TPPMA was determined to be ~20, ~25 and ~3 based on the ^{1}H NMR analysis, respectively (Figure S11).

ROS generation

9,10-Anthracenediyl-bis(methylene)dimalonic acid (ABDA) was used as the indicator of the generation of $^1O_2$. Micelles (TPP concentration, 15 μg/mL) and ABDA (60 μg/mL) were dispersed in PBS. Subsequently, the absorbance intensity of the mixture was monitored after irradiated by near infrared light (640-660 nm, 200 mW/cm^2) every 1 min.

Cellar uptake

4T1 cells were inoculated in confocal dish at a density of 1 × 10^6 cells per dish and cultured overnight. Then the 4T1 cells were treated by micelles (TPP-equivalent concentration, 15 μg/mL) and incubated for 2, 6, or 12 h. After that, the cells were washed with PBS twice and incubated with 4% paraformaldehyde for 15 min. The cell nuclei were stained with 4′,6-diamidino-2-phenylindole dihydrochloride (DAPI) for 25 min. Finally, the cells were observed by confocal laser scanning microscope (CLSM).

For flow cytometry analysis, 4T1 cells were inoculated in 12-well plates at a density of 1 × 10^6 cells per well and cultured overnight. Then the 4T1 cells were treated

by micelles (TPP-equivalent concentration, 15 μg/mL) and incubated for 1, 2, 4, or 6 h. After that, the cells were washed with PBS twice and incubated with trypsin for 1 min. Finally, the cells were re-suspended in PBS and measured by flow cytometry.

Intracellular ROS generation

4T1 cells were inoculated in confocal dish at a density of 1 × 10^6 cells per dish and cultured overnight. Then the 4T1 cells were treated by micelles (TPP concentration, 15 μg/mL) and incubated for 8 h. After that, the cells were stained with 2',7'-dichlorodihydrofluorescein diacetate (DCFH-DA) for 30 min. Afterward, the 4T1 cells were irradiated with near infrared light for 10 min. Then the cells were observed by CLSM.

Cytotoxicity

4T1 cells were inoculated in 96-well plates at a density of 1 × 10^4 cells per well and cultured overnight. Then the 4T1 cells were treated by micelles (TPP concentration, 15 μg/mL) and incubated for 8 h. After that, the 4T1 cells were irradiated with near infrared light for 10 min and further incubated for 16 h. Finally, the cells were cultured with cell counting kit-8 (CCK-8, 100 μL) for 1 h. The absorbance at 450 nm were measured by microplate reader. The relative cell viability was calculated as follows: cell viability = (absorbance in treated group/absorbance in the control group) × 100%.

Annexin V-FITC/propidium iodide (PI) staining

4T1 cells were inoculated in 12-well plates at a density of 1×10^6 cells per well and cultured overnight. Then the 4T1 cells were treated by micelles (TPP concentration, 15 μg/mL) and incubated for 8 h. After that, the cells were irradiated with near infrared light for 10 min and further incubated for 16 h. Subsequently, the cells were collected and resuspended in Annexin V-FITC/PI, and further incubated for 20 min. Finally, the cells were measured by flow cytometry.

In vitro IDO staining

4T1 cells were incubated in confocal dish at a density of 1×10^6 cells per dish and cultured overnight. The cells were treated with IFN-γ (100 ng/mL) and the micelles (SR@PMT or SR@ET-PMT) with or without irradiation by the red LED light (640-660 nm) at an intensity of 200 mW/cm^2 for 10 min. After incubation for 24 h, the cells were washed with PBS twice and incubated with 4% paraformaldehyde for 15 min. Then the cells were incubated with immunostaining blocking buffer for 60 min. After that, the cells were washed with PBS thrice and incubated with IDO1 antibody overnight at 4 ºC. Whereafter, the cells were washed thrice and incubated with Alexa Flour 488-labeled Goat Anti-Rabbit lgG(H+L) for 60 min. And the cell nuclei were stained with DAPI for 25 min. Then the cells were observed by CLSM.

In vitro STING activation

Bone-marrow derived dendritic cells (BMDCs) were isolated from BALB/c mice. To study the ability of micelles to trigger DC maturation, the collected DCs were seeded in 6-well plates and cultured for 24 h. Then the cells were incubated with PBS solutions containing SR-717, SR@PMT, or SR@ET-PMT at the SR-equivalent concentration of 10 μg/mL with or without light-irradiation pretreatment. After incubation for another 24 h, the cells were collected and stained with CD11c, CD86, and CD80 for 30 min. After that, the cells were measured by flow cytometry. Meanwhile, the proinflammatory cytokines (IFN-β) and IL-6 from BMDCs suspensions were measured by using ELISA kits according to a standard protocol.

Tumor models and in vivo fluorescence imaging

4T1 cells (1×10^6) were subcutaneously injected into left flanks of BALB/c mice. When the tumor volume reached ~ 100 mm^3, the mice were injected intravenously with the micelles (DiR@PMT or DiR@ET-PMT) loading 1,1'-dioctadecyl-3,3,3',3'-tetramethylindotricarbocyanine iodide (DiR) at the DiR-equivalent dose of 0.5 mg/kg body weight. Then the fluorescence signals over time were monitored by in vivo imaging system (IVIS).

Characterization

All ^{1}H NMR spectra was recorded on a Bruker AV400 NMR 400MHz spectrometer using $CDCl_3$, D_2O or DMSO-d_6 as the solvent. UV-vis spectra were recorded on a

Shimadzu UV-3600 spectrophotometer. Particle sizes, and particle size distributions were measured on a zeta potential analyzer with dynamic laser light scattering (DLS) equipped a Malvern Zeta sizer Nano ZS90, a He-Ne laser (633 nm), and 173° collecting optics. Transmission electron microscope (TEM) measurements were conducted on a Hitachi 7650 electron microscope at the acceleration voltage of 100 kV. The sample solutions are dropped onto a copper grid, uranium acetate solution (10 μL, 2wt%) is mixed with the sample solution. After 10 min, the excess solution was removed and the sample was dried at room temperature overnight. The confocal laser scanning microscope (CLSM) image s were obtained on an FV1000 (Olympus Company, Japan). Flow cytometry analysis for cell-apoptosis analysis was conducted by a BD LSRFortessa flow cytometry (Becton, Dickinson and Company, USA).

2. Supporting Figures

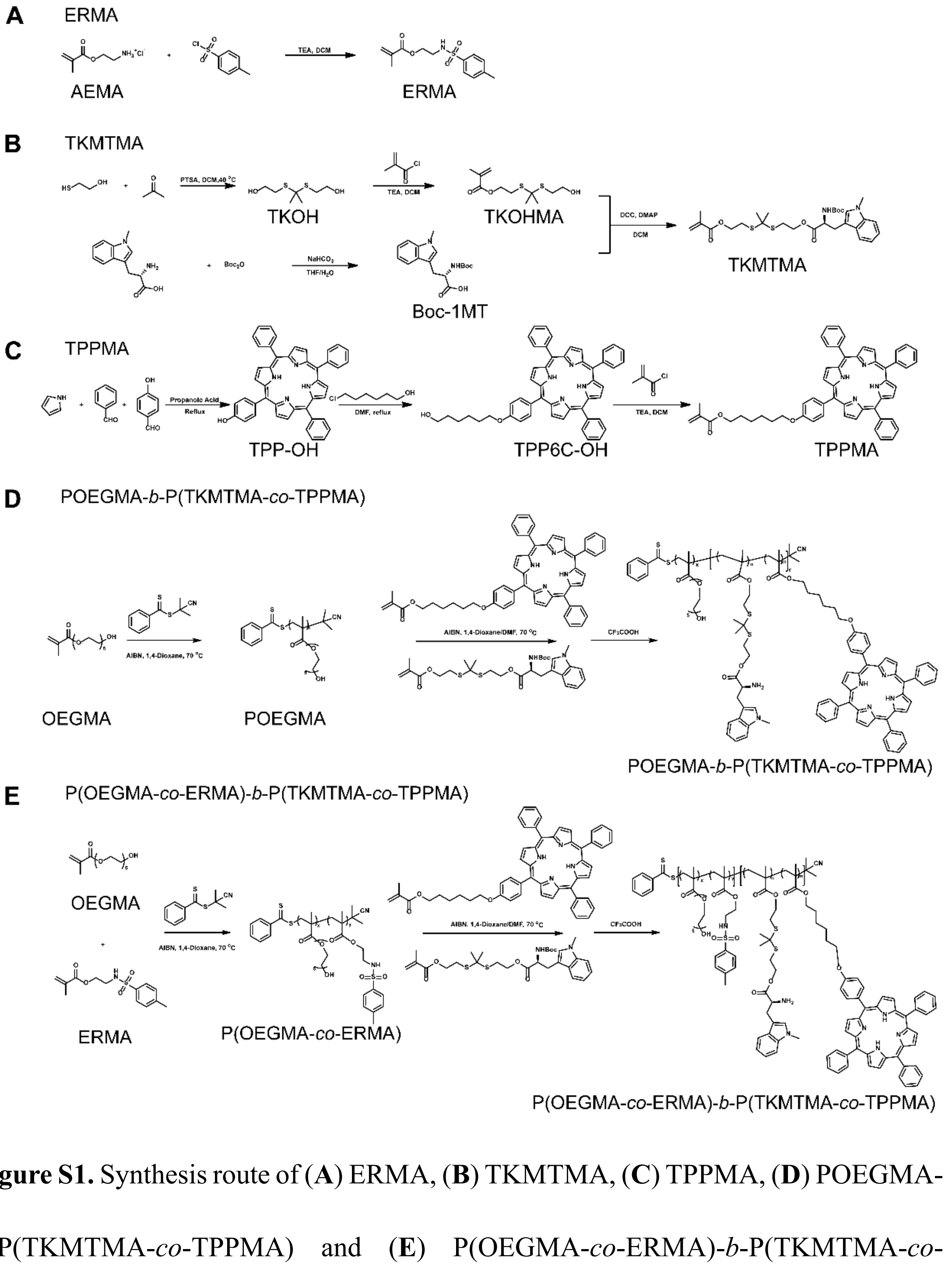

Figure S1. Synthesis route of (**A**) ERMA, (**B**) TKMTMA, (**C**) TPPMA, (**D**) POEGMA-*b*-P(TKMTMA-*co*-TPPMA) and (**E**) P(OEGMA-*co*-ERMA)-*b*-P(TKMTMA-*co*-TPPMA).

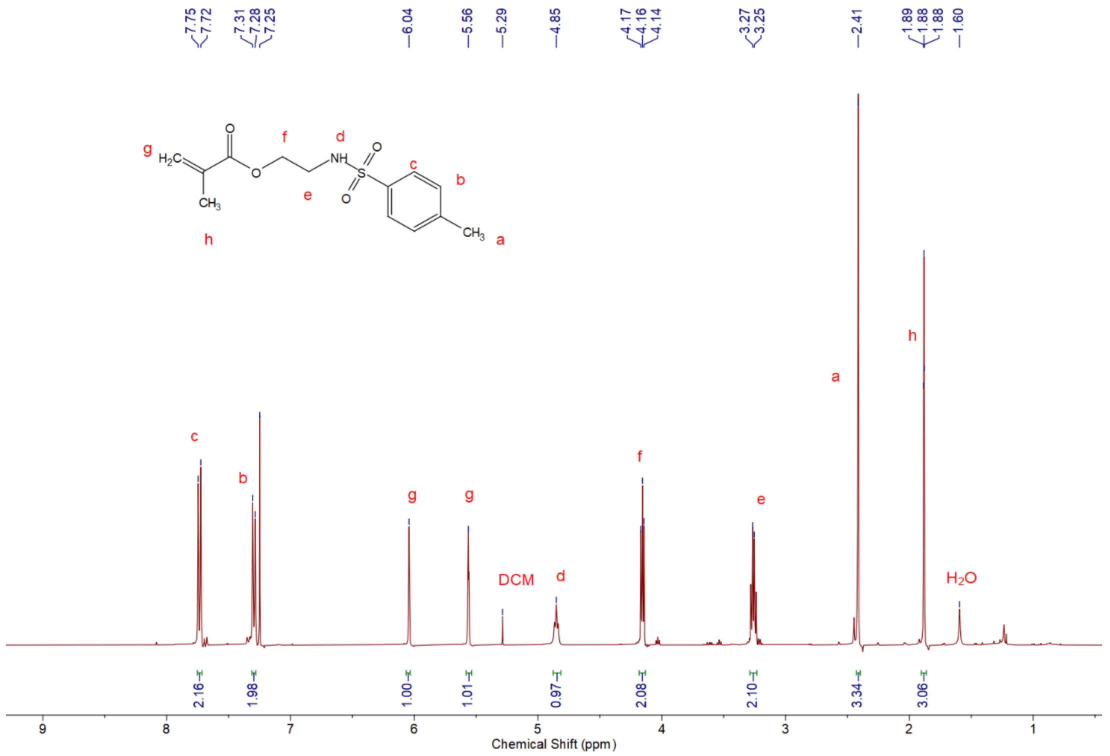

Figure S2. ^{1}H NMR spectrum recorded for ERMA in $CDCl_3$.

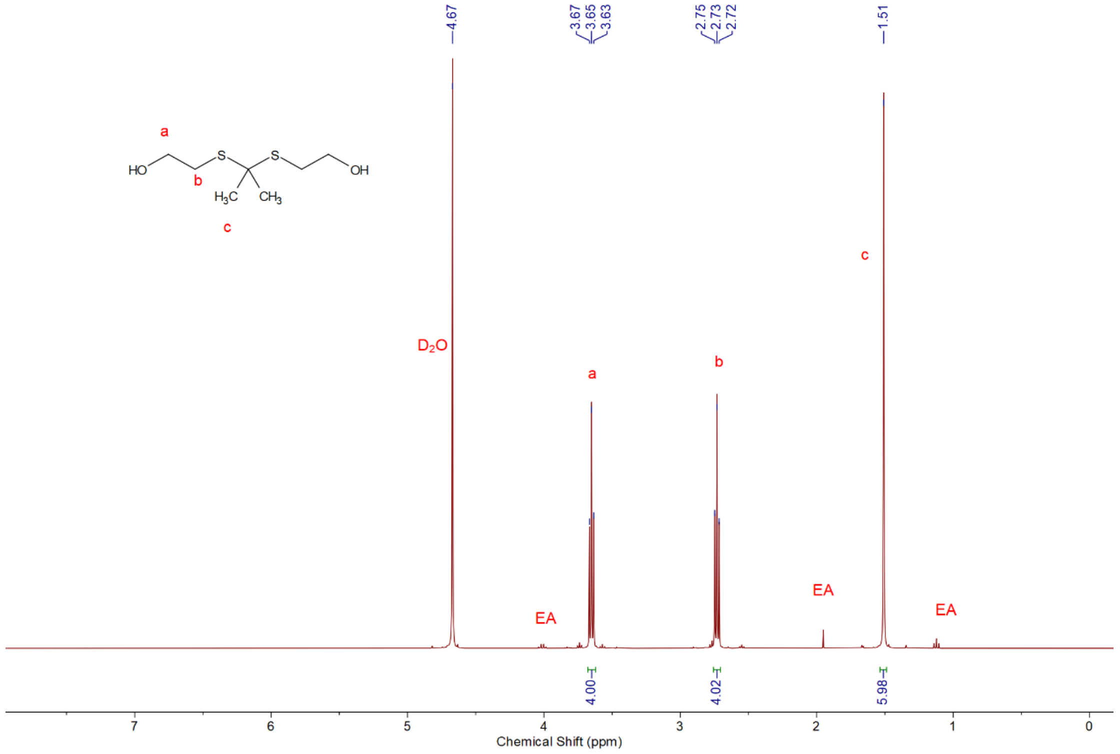

Figure S3. ^{1}H NMR spectrum recorded for TKOH in D_2O.

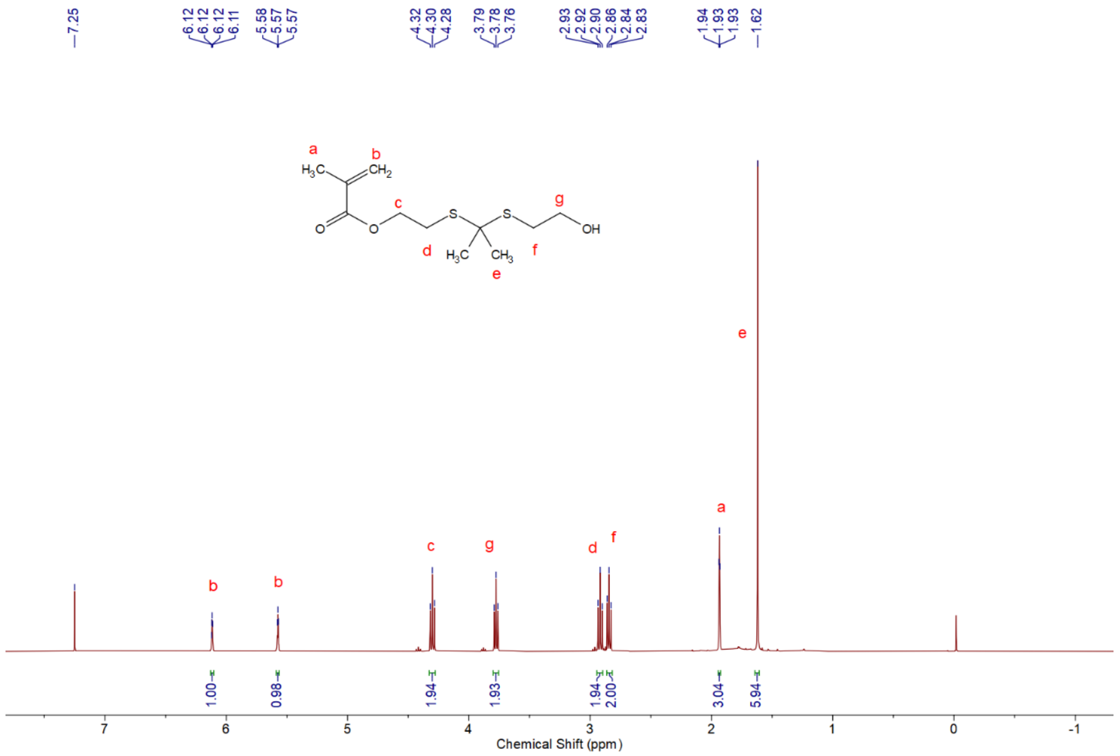

Figure S4. ^1H NMR spectrum recorded for TKOHMA in $CDCl_3$.

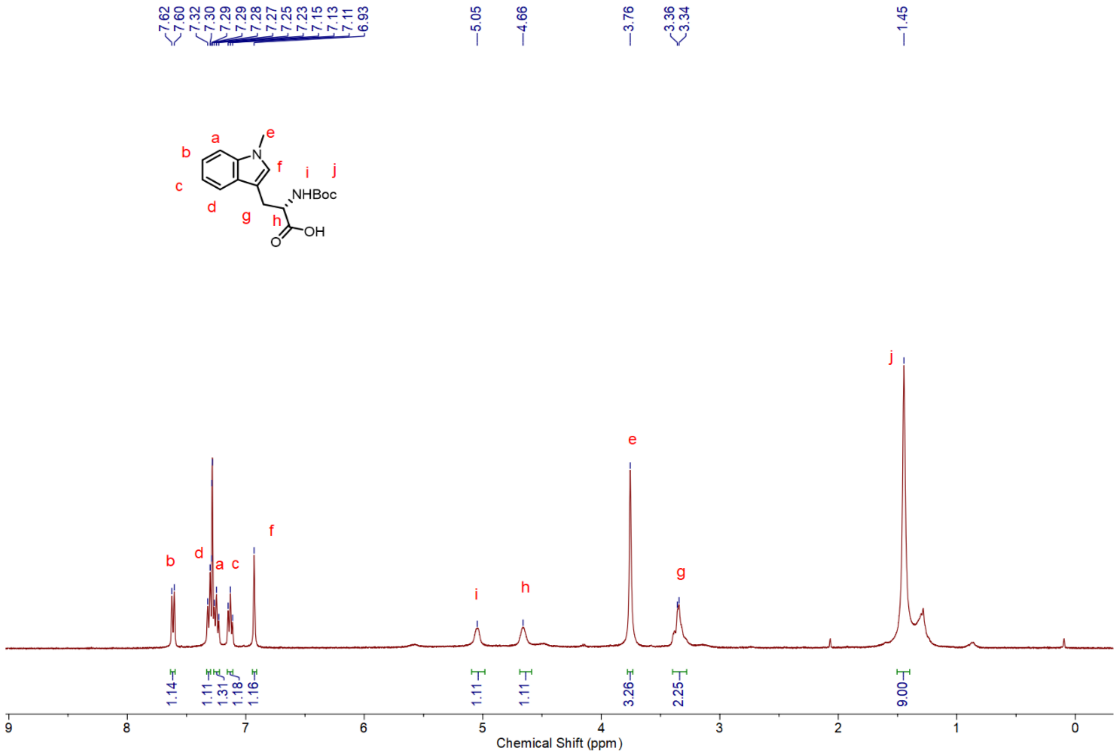

Figure S5. ^1H NMR spectrum recorded for Boc-1MT in $CDCl_3$.

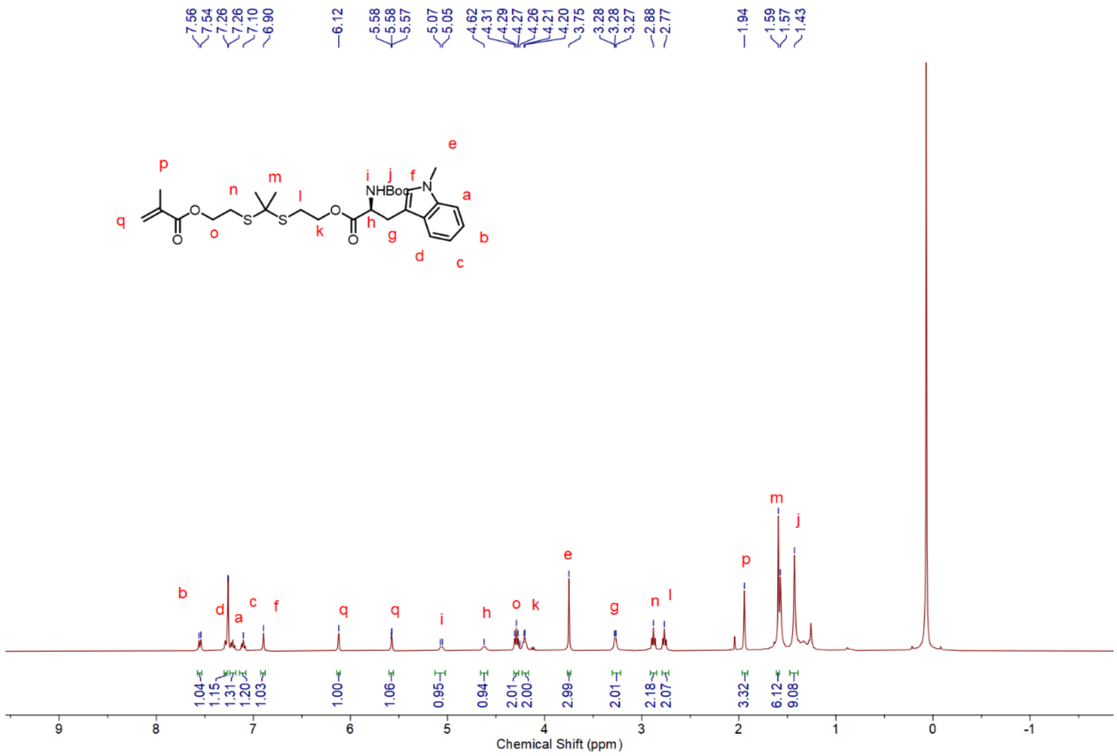

Figure S6. ^{1}H NMR spectrum recorded for TKMTMA in $CDCl_3$.

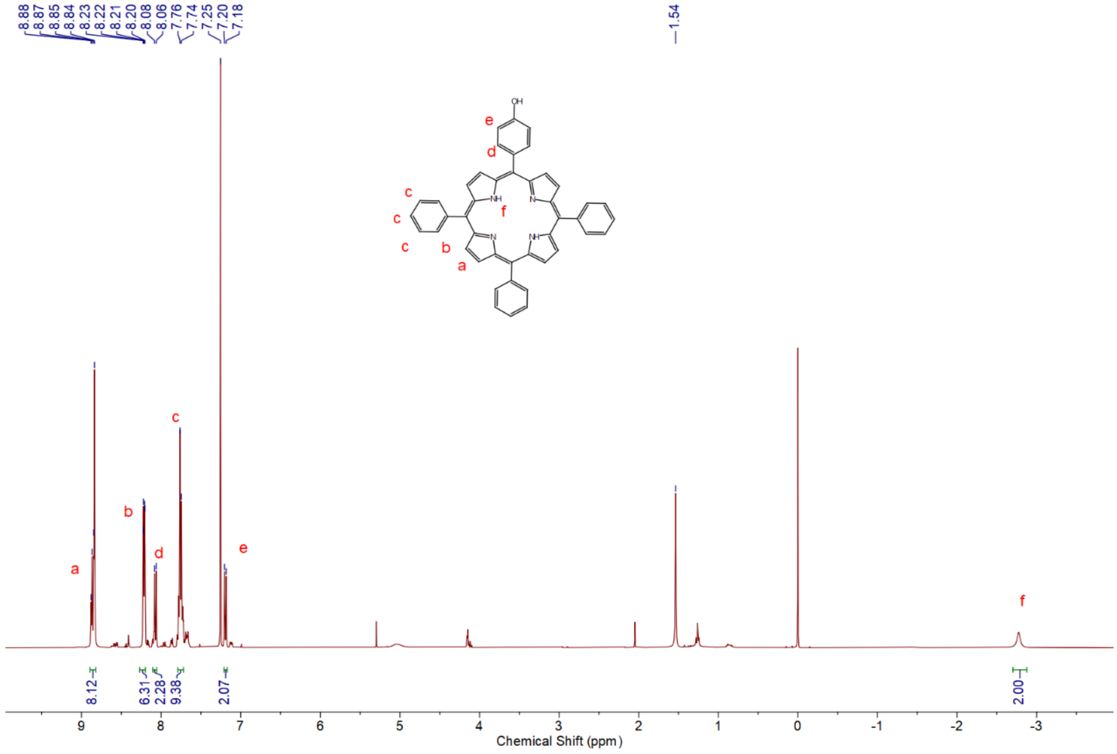

Figure S7. ^{1}H NMR spectrum recorded for TPP-OH in $CDCl_3$.

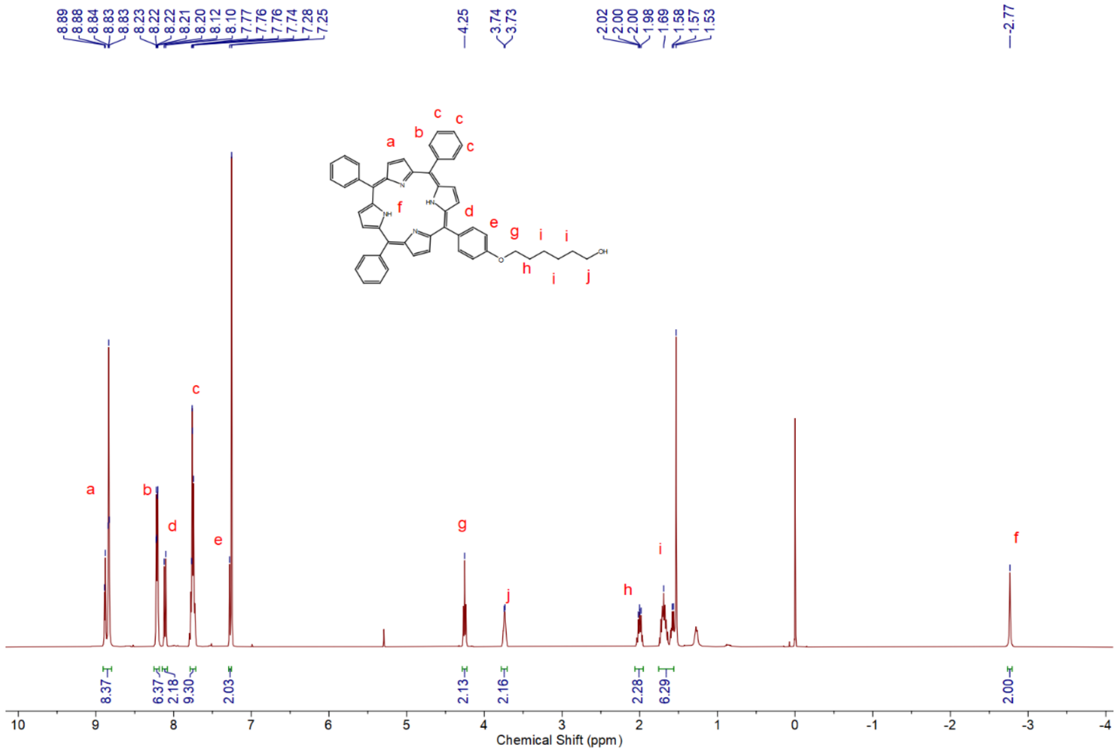

Figure S8. ^{1}H NMR spectrum recorded for TPP6C-OH in $CDCl_3$.

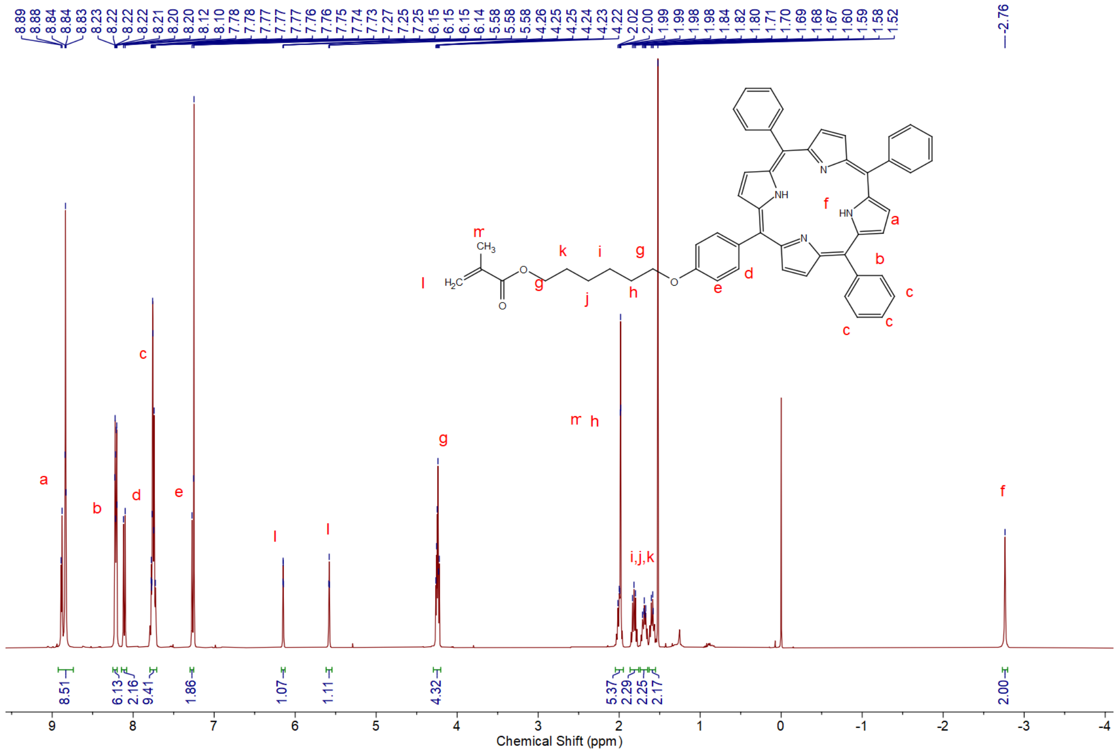

Figure S9. ^{1}H NMR spectrum recorded for TPPMA in $CDCl_3$.

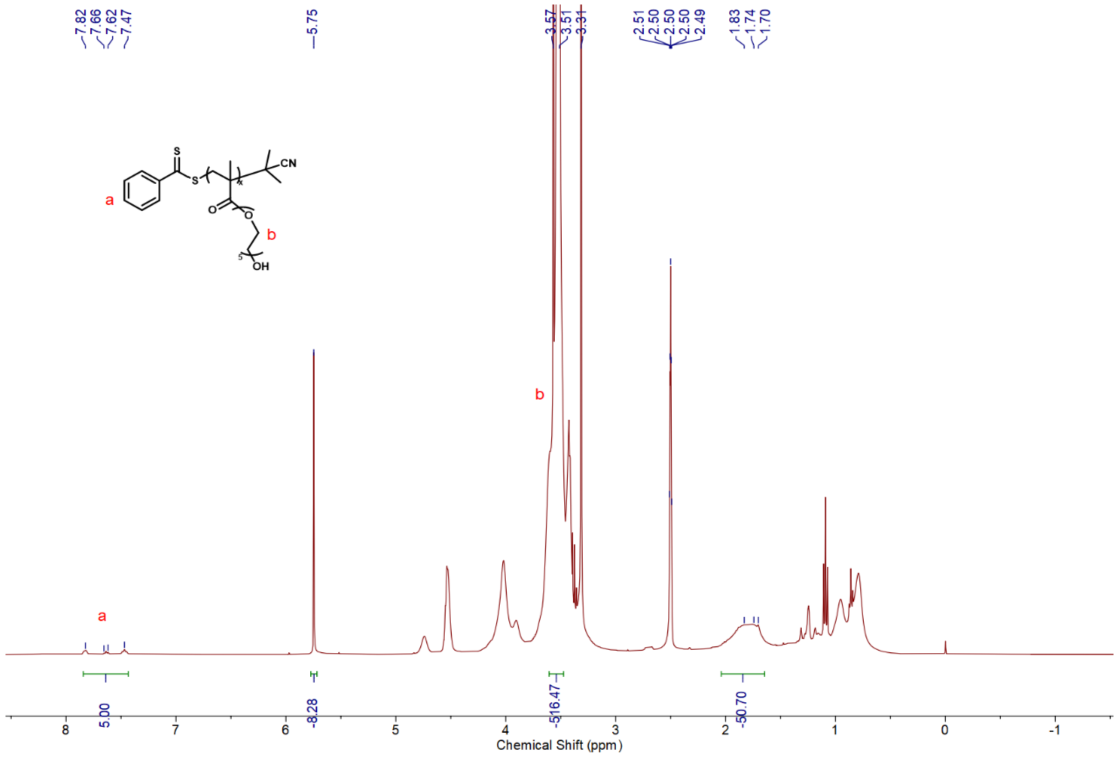

Figure S10. ^{1}H NMR spectrum recorded for POEGMA in DMSO-d_6.

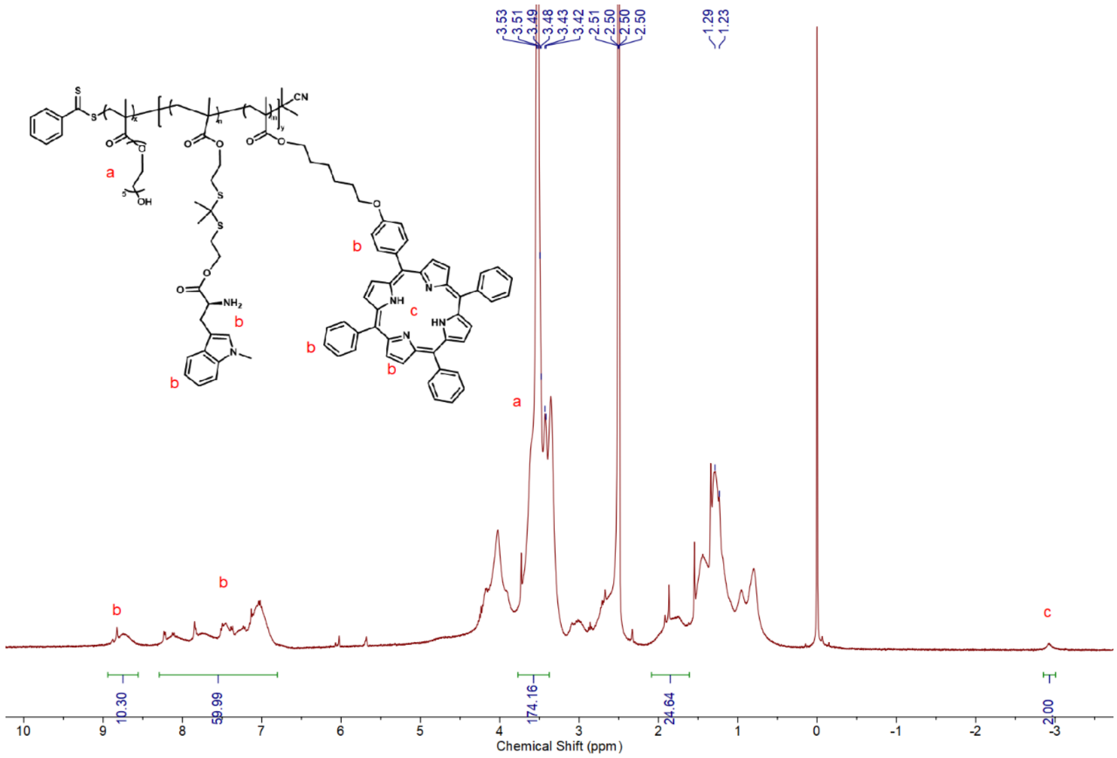

Figure S11. ^{1}H NMR spectrum recorded for POEGMA-*b*-P(TKMTMA-*co*-TPPMA) in DMSO-d_6.

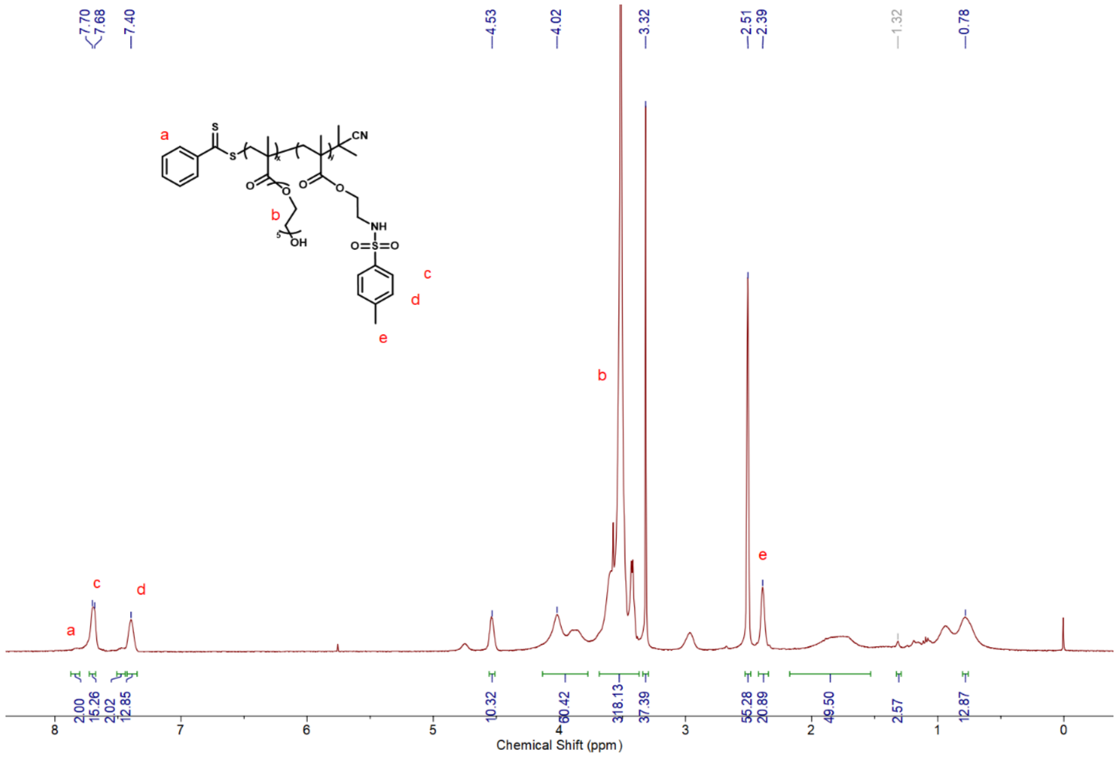

Figure S12. ^{1}H NMR spectrum recorded for P(OEGMA-*co*-ERMA) in DMSO-d_6.

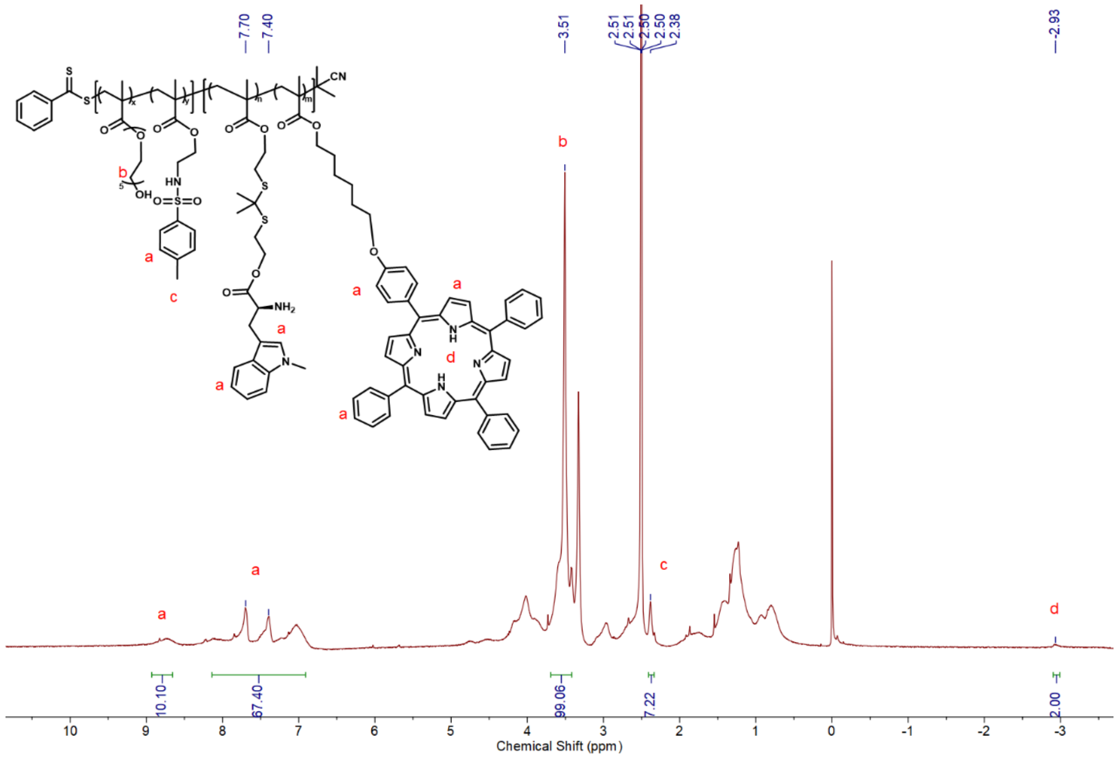

Figure S13. ^{1}H NMR spectrum recorded for P(OEGMA-co-ERMA)-*b*-P(TKMTMA-*co*-TPPMA) in DMSO-d_6.

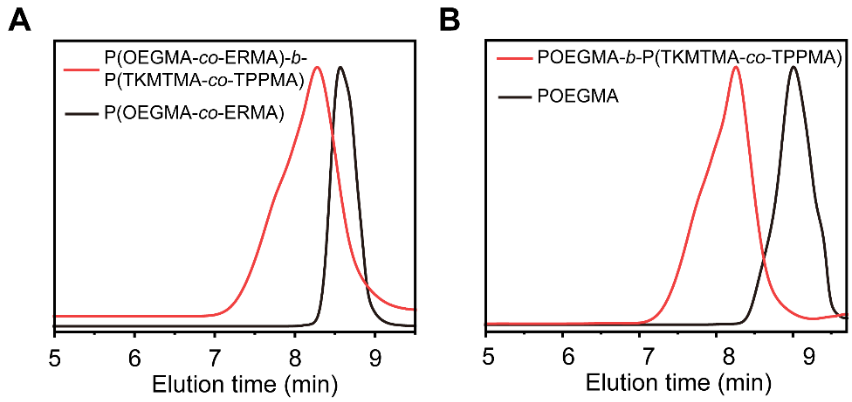

Figure S14. (**A**) GPC traces recorded for P(OEGMA-c*o*-ERMA) (M_n = 7000 Da, M_w/M_n = 1.04) and P(OEGMA-c*o*-ERMA)-*b*-P(TKMTMA-*co*-TPPMA) (M_n = 22000 Da, M_w/M_n = 1.23). (**B**) GPC traces recorded for POEGMA (M_n = 6000 Da, M_w/M_n = 1.12) and POEGMA-*b*-P(TKMTMA-*co*-TPPMA) (M_n = 20000 Da, M_w/M_n = 1.18).

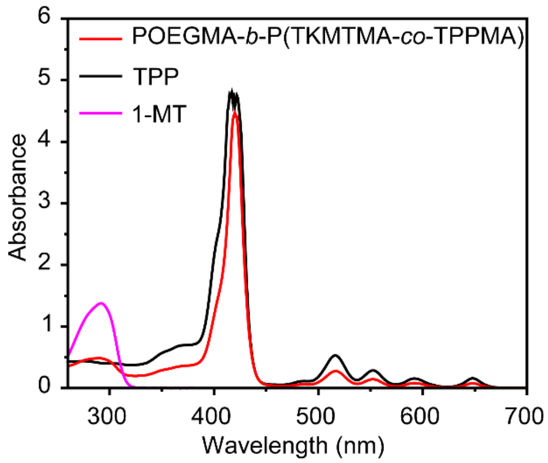

Figure S15. UV-vis absorbance spectra of 1-MT, TPP and POEGMA-*b*-P(TKMTMA-*co*-TPPMA) in chloroform.

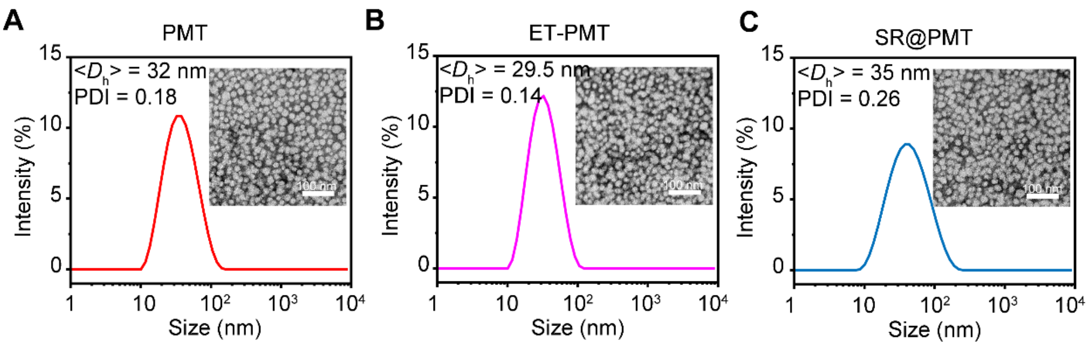

Figure S16. (**A**) Size distribution and TEM images of PMT. Scale bar = 100 nm. (**B**) Size distribution and TEM images of ET-PMT. Scale bar = 100 nm. (**C**) Size distribution and TEM images of SR@PMT. Scale bar = 100 nm.

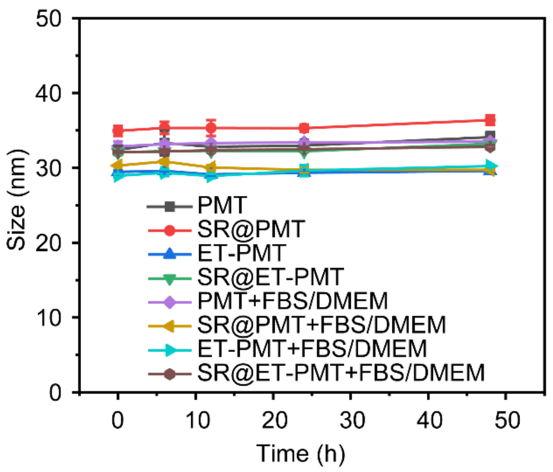

Figure S17. Time-dependent size change of PMT, SR@PMT, ET-PMT, and SR@ET-PMT in PBS or DMEM with 10% FBS by DLS analysis.

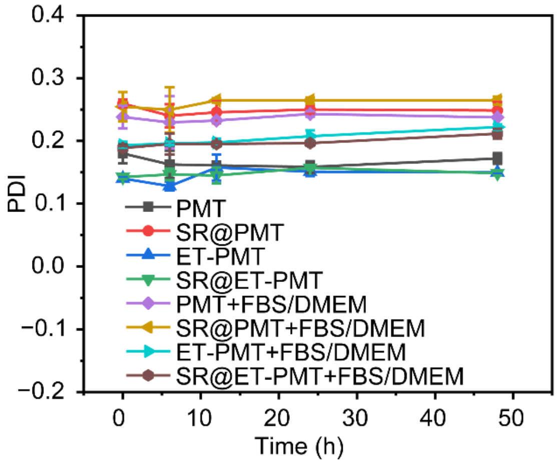

Figure S18. Time-dependent polydispersity index (PDI) of PMT, SR@PMT, ET-PMT, and SR@ET-PMT in PBS or DMEM with 10% FBS by DLS analysis.

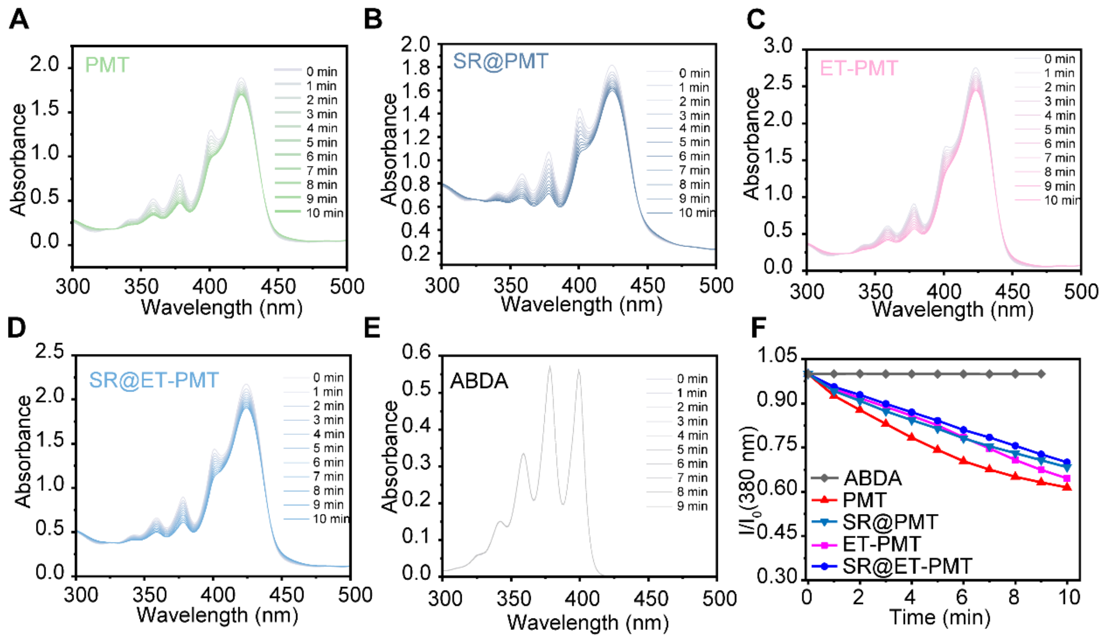

Figure S19. (**A-E**) Absorbance spectra of PMT, SR@PMT, ET-PMT, SR@ET-PMT and ABDA after incubation with ABDA under light irradiation at different times. (**F**) Changes in the absorption values at 380 nm of ABDA in the presence of PMT, SR@PMT, ET-PMT, and SR@ET-PMT.

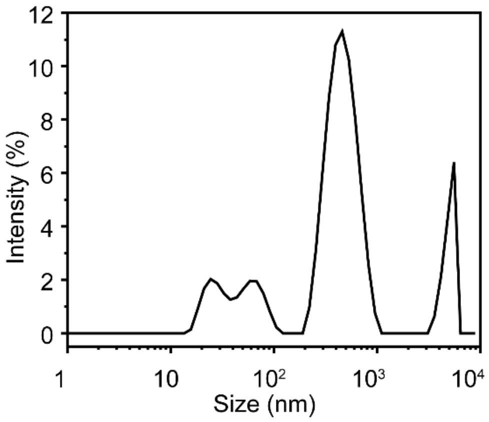

Figure S20. Size distribution of SR@PMT after 15 min of light irradiation.

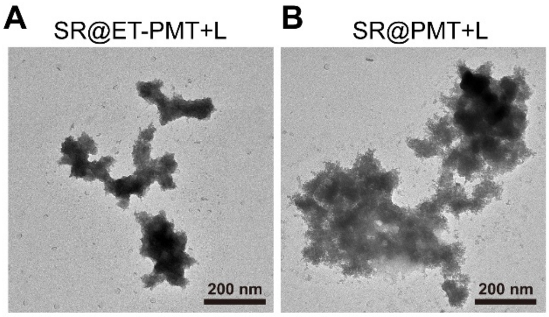

Figure S21. TEM images of (**A**) SR@ET-PMT and (**B**) SR@PMT after 15 min of light irradiation. Scale bar = 200 nm.

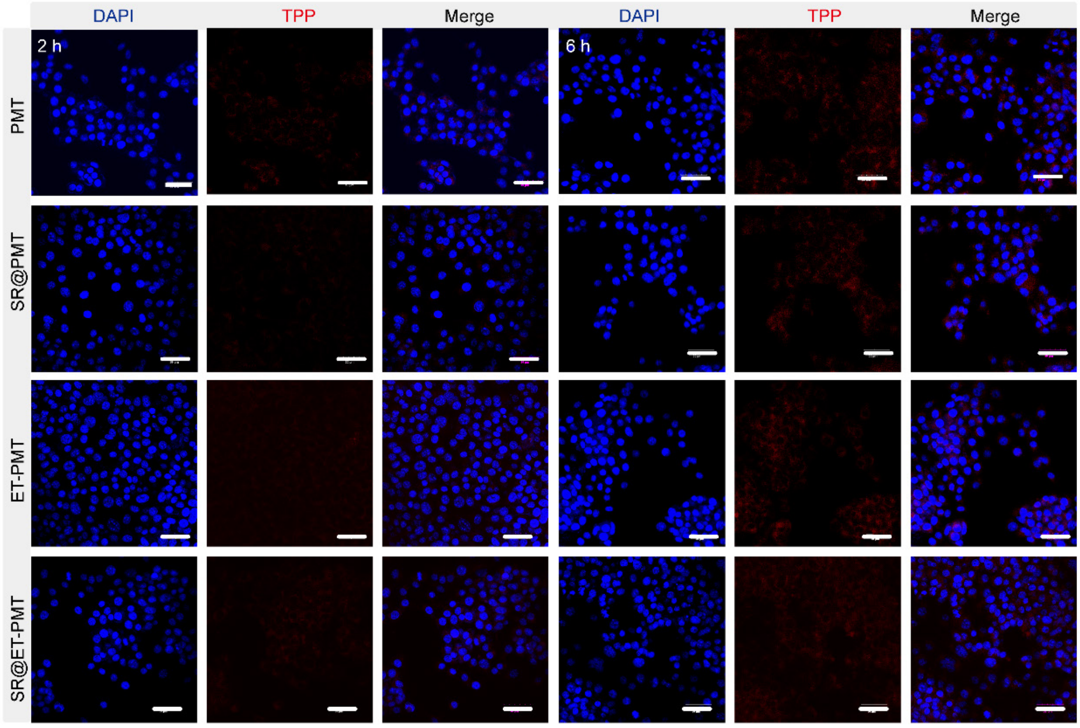

Figure S22. CLSM images of 4T1 cells after incubation with PMT, SR@PMT, ET-PMT, and SR@ET-PMT for different times. Scale bars are 50 µm.

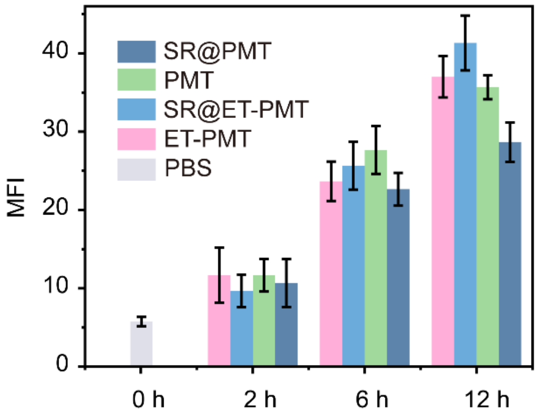

Figure S23. Quantitative analysis of mean TPP fluorescence intensity (MFI) of 4T1 cells after incubation with PMT, SR@PMT, ET-PMT, and SR@ET-PMT (TPP concentration, 15 µg/mL) for 2, 6, and 12 h. Mean ± s.d., n = 3.

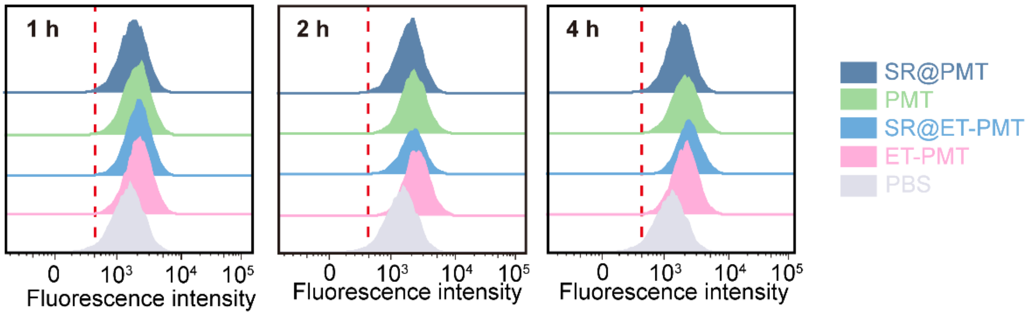

Figure S24. Flow cytometry analysis of 4T1 cells after incubation for different times.

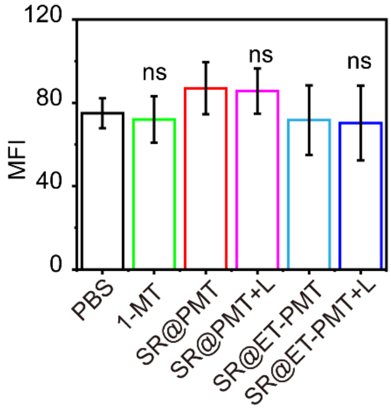

Figure S25. Quantitative analysis of mean IDO fluorescence intensity (MFI) of 4T1 cells after different treatments. Mean ± s.d., n = 3.

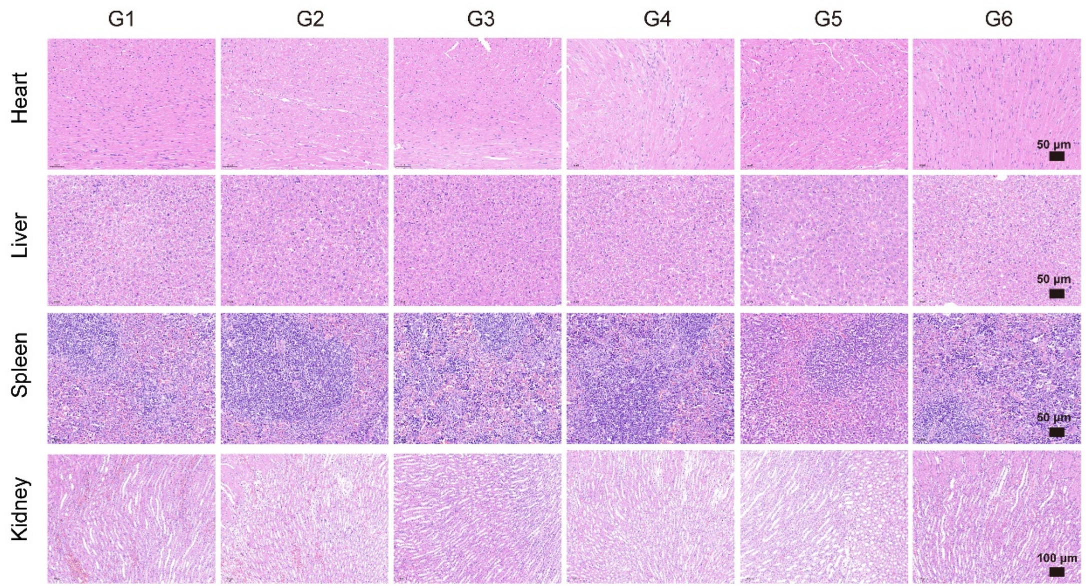

Figure S26. H&E staining images of major organs in 4T1 tumor-bearing mice after treatments.

3. References

1. W. Wang, Y. Zhang, Z. Wang, X. Liu, S. Lu, X. Hu. A native drug-free macromolecular therapeutic to trigger mutual reinforcing of endoplasmic reticulum Stress and mitochondrial dysfunction for cancer treatment. ACS Nano. 2023; 17: 11023-11038.
2. S. G. Awuah, Y.-R. Zheng, P. M. Bruno, M. T. Hemann, S. J. Lippard. A Pt(IV) pro-drug preferentially targets indoleamine-2,3-dioxygenase, providing enhanced ovarian cancer Immuno-chemotherapy. J Am Chem Soc. 2015; 137: 14854-7.
3. X. Shen, Z. Zhou, D. Qi, Y. Li, Z. Zeng, X. He, X. Xia, C. Zhu, X. Feng. Highly uniform self-assembled microspheres from single macromolecule self-recognition for enhanced cancer immunotherapy. Chem Commun. 2020; 56: 9190-3.
4. S. Zhong, C. Chen, G. Yang, Y. Zhu, H. Cao, B. Xu, Y. Luo, Y. Gao, W. Zhang, Acid-triggered nanoexpansion polymeric micelles for enhanced photodynamic therapy. ACS Appl Mater Interfaces. 2019; 11: 33697-33705.
